# Parameter Estimation for Gene Regulatory Networks from Microarray Data: Cold Shock Response in ***Saccharomyces cerevisiae***

**DOI:** 10.1007/s11538-015-0092-6

**Published:** 2015-09-29

**Authors:** Kam D. Dahlquist, Ben G. Fitzpatrick, Erika T. Camacho, Stephanie D. Entzminger, Nathan C. Wanner

**Affiliations:** Department of Biology, Loyola Marymount University, 1 LMU Drive, MS 8888, Los Angeles, CA 90045 USA; Department of Mathematics, Loyola Marymount University, 1 LMU Drive, UH 2700, Los Angeles, CA 90045 USA; School of Mathematical and Natural Sciences, Arizona State University, Mail Code 2352, P.O. Box 37100, Phoenix, AZ 85069-7100 USA

**Keywords:** Dynamic network model, Penalized least squares

## Abstract

We investigated the dynamics of a gene regulatory network controlling the cold shock response in budding yeast, *Saccharomyces cerevisiae*. The medium-scale network, derived from published genome-wide location data, consists of 21 transcription factors that regulate one another through 31 directed edges. The expression levels of the individual transcription factors were modeled using mass balance ordinary differential equations with a sigmoidal production function. Each equation includes a production rate, a degradation rate, weights that denote the magnitude and type of influence of the connected transcription factors (activation or repression), and a threshold of expression. The inverse problem of determining model parameters from observed data is our primary interest. We fit the differential equation model to published microarray data using a penalized nonlinear least squares approach. Model predictions fit the experimental data well, within the 95 % confidence interval. Tests of the model using randomized initial guesses and model-generated data also lend confidence to the fit. The results have revealed activation and repression relationships between the transcription factors. Sensitivity analysis indicates that the model is most sensitive to changes in the production rate parameters, weights, and thresholds of Yap1, Rox1, and Yap6, which form a densely connected core in the network. The modeling results newly suggest that Rap1, Fhl1, Msn4, Rph1, and Hsf1 play an important role in regulating the early response to cold shock in yeast. Our results demonstrate that estimation for a large number of parameters can be successfully performed for nonlinear dynamic gene regulatory networks using sparse, noisy microarray data.

**Electronic supplementary material:**

The online version of this article (doi:10.1007/s11538-015-0092-6) contains supplementary material, which is available to authorized users.

## Introduction

All organisms must respond to changes and stresses in their environment to survive and reproduce. Such environmental stresses include changes in nutrient or oxygen availability, changes in osmolarity, salinity, or pH, the presence of reactive oxygen species or other damaging agents, and sudden or large changes in temperature, either an increase (heat shock) or decrease (cold shock). Organisms respond to environmental stresses through characteristic programs of gene expression. Among the most interesting and challenging problems in understanding this environmental stress response is the dynamic behavior of gene expression networks within the cell. The careful regulation of these networks is a fundamental activity of the organism. In this paper, we discuss the development and application of a dynamical systems model for regulation of gene expression during the early response to cold shock in budding yeast.

Our focus on *Saccharomyces cerevisiae* and cold shock is motivated by a number of factors. These yeast have been studied extensively, especially their response to heat shock, which occurs through the induction of heat shock proteins (Morano et al. 2012). These heat shock proteins are universally conserved across all organisms and have been very well characterized. However, the response to cold shock has been less well studied, although its effects on cellular physiology are known (Thieringer et al. 1998; Al-Fageeh and Smales 2006; Aguilera et al. 2007). Decreases in temperature cause a reduction in membrane fluidity, a reduction in enzymatic activity, the stabilization of DNA and RNA secondary structures, and the impairment of protein synthesis. Similarly to heat shock, cold shock does induce the expression of a set of “cold shock” proteins; however, these proteins are not universally conserved. Much remains to be discovered about the molecular mechanisms and regulation of the response to cold temperatures in yeast. The model we develop provides some new tools for investigating the regulation of this response and provides new biological insight into this phenomenon.

Biologically, computationally, and mathematically, parameter estimation remains a significant challenge for the modeling of gene regulatory dynamics, even for medium-scale networks of just 5–10 interacting genes, (Cao and Zhao 2008; Lillacci and Khammash 2010; Kuwahara et al. 2013; Fan et al. 2015). The large number of parameters, the highly nonlinear dynamics of gene regulation, and the noisiness and relative sparseness of time course microarray data make parametric inference a difficult problem requiring mathematical and numerical care. Our approach integrates numerical solution of the ODE model, state-of-the-art optimization algorithms, and novel use of penalization to infer parameters for a relatively large network with few temporal data points. Our results demonstrate that large-scale parameter estimation can be successfully performed for nonlinear dynamic gene regulatory networks using sparse, noisy microarray data.

Our model involves a few key ingredients. One is a network of transcription factors that activate or repress transcription of genes needed for the cell to respond to the cold shock stress. The network itself can be thought of as a simple qualitative model in its own right, and many investigators have explored the problem of network inference from gene expression data (for a review see Hecker et al. 2009 and references therein). Instead, we start with an experimentally defined network so that we can take the next step of developing quantitative production and degradation dynamics for the transcription factors involved in the cold shock response.

We then develop parameter estimation techniques for extracting rate parameter information from time course microarray data obtained from cold shock experiments to infer the direction (activation or repression) and magnitude of influence that regulatory transcription factors have on their target genes. Other models of this type have either been developed on relatively simple small gene circuits (e.g., Cao and Zhao 2008) or have used data from biological systems that are already well understood (e.g., the yeast cell cycle, Vu and Vohradsky 2007), so little new biological insight is gained. The novelty of our approach is to take a problem where relatively little is known about the biology and create a meaningful dynamical model of the system. A number of methods have been proposed and implemented for fitting differential equation models to data (see, e.g., Cao and Zhao 2008, for an excellent review). In this paper, we discuss a penalized nonlinear least squares approach to parameter estimation, which we have applied with success to a number of problems, ranging from the dynamics of college drinking (Ackleh et al. 2009) and subsurface contaminant transport (Bailey and Fitzpatrick 1997) to inverse interferometry (Fitzpatrick and Keeling 1997) and liquid chromatography (Fitzpatrick 1993). This approach has largely been avoided in gene regulatory models due to its mathematical and numerical complexity. The advantages of our approach over extended Kalman filtering (Lillacci and Khammash 2010; Fan et al. 2015) or profiling methods (Cao and Zhao 2008) is that appropriate treatment of the penalized least squares allows the estimation of a fairly high-dimensional parameter from relatively sparse temporal data, a common challenge with microarrays and other measurement technologies. Here we compare the solution of the differential equations to microarray data from cold shock experiments on *S. cerevisiae, *using penalized least squares in an innovative way, to extract parameter estimates and determine the regulatory directions (activation or repression) and the strengths of the regulatory relationships of controlling genes on targets in a complex feedback network of 21 genes (nodes) and 31 regulatory relationships (edges).

The paper is organized as follows. In Sect. 2, we describe the model organism *S. cerevisiae*, the environmental stress of cold shock, and the determination of a regulatory network structure. The nature of the microarray data that we use for parameter estimation is discussed in Sect. 3, while Sect. 4 is devoted to the mathematical model and the estimation problem. Section 5 provides the results of our parameter estimation process. We close the paper in Sect. 6 with some concluding remarks that discuss the results and suggest future directions.

## Regulation of the Response to Cold Shock in ${\mathbf {S. cerevisiae}}$

As a single-celled eukaryote, budding yeast, *Saccharomyces cerevisiae*, must respond to changes and stresses in the environment such as changes in nutrient or oxygen availability, changes in osmolarity, salinity, or pH, the presence of reactive oxygen species or other damaging agents, and sudden or large changes in temperature, either an increase (heat shock) or decrease (cold shock; Dawes 2004). Yeast respond to environmental stresses through characteristic programs of gene expression, called the Environmental Stress Response (ESR; Gasch et al. 2000; Causton et al. 2001). With the advent of high-throughput, whole-genome methods such as DNA microarrays, programs of gene expression, including the ESR, have been elucidated as never before. These data are key to developing a fundamental understanding of cell function. Mechanistic models of gene regulatory networks that have been validated by experiment can then yield additional insights. This paper details modeling and parameter estimation for a gene regulatory network controlling the cold shock response in yeast.

Unlike the response to heat shock and other environmental stresses, the transcriptional response to cold shock has been relatively less well studied in yeast. The previous studies that exist have revealed that the response varies depending on the temperature and the length of time spent at the cold temperature. The cold shock response occurs between the temperatures of 10 and $18\,^{\circ }\hbox {C}$ (Sahara et al. 2002; Schade et al. 2004; Tai et al. 2007), and the near-freezing response occurs between 0 and $10\,^{\circ }\hbox {C}$ (Kandror et al. 2004; Murata et al. 2006). The early response occurs after 10 min up to 2 h of cold temperatures, and the late response occurs after 12 h of cold or near-freezing temperatures (Kandror et al. 2004; Schade et al. 2004), although the exact transition time between the early and late responses has not been definitively determined. However, it is clear from these studies that the early and late responses represent two different biological phenomena of first adaptation by the cells to the cold temperature, followed by acclimation. These two distinct processes require the expression of different sets of genes and different sets of regulatory transcription factors to regulate them. Indeed, these studies revealed that the cold shock late response, but not the early response, include the ESR genes induced by many environmental stresses. Through the use of gene deletion experiments, Schade et al. (2004) and Kandror et al. (2004) also determined that the ESR genes in the late response to cold and near-freezing temperatures, respectively, were regulated by the Msn2 and Msn4 transcription factors, as they are during other environmental stresses. However, the transcription factors responsible for the induction of the early response genes and the overall regulatory mechanism governing this early response remain largely unknown. Furthermore, there is ample evidence to suggest that environmental stress response pathways overlap, as is seen by the induction of the same set of ESR genes under multiple stress conditions (Gasch et al. 2000; Causton et al. 2001). Finally, DNA microarray experiments comparing gene expression changes when the Leu3 transcription factor was deleted or overexpressed has revealed that many genes that are not direct targets of that factor were affected in the experiment due to indirect effects (Tang et al. 2006). These indirect effects are most likely due to regulatory relationships between transcription factors. Thus, these questions remain: (1) which transcription factors control the early response to cold shock in *S. cerevisiae*? (2) what is the extent of ESR pathway overlap? (3) which part of the early transcriptional response to cold shock is due to indirect effects of other transcription factors? To approach these questions, we need complementary types of high-throughput genomic data, the tools of mathematical biology, and the perspective of systems biology.

**Fig. 1:**
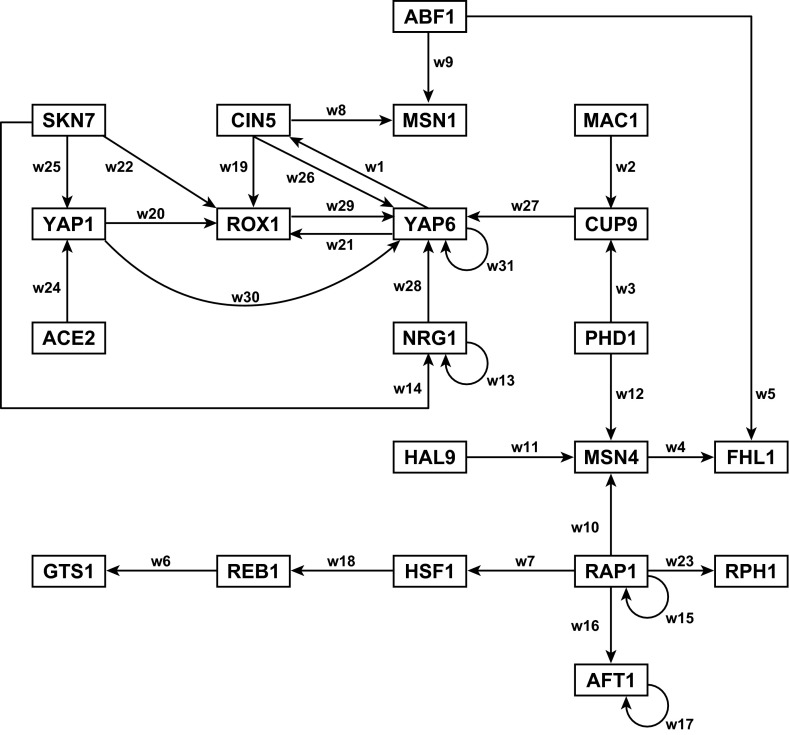
Cold shock gene regulatory network diagram. The *arrows* indicate the direction of regulation (transcription factor to target) and do not represent activation here. *Each edge* is annotated with the weight parameter index from Table 5, referred to in Figs. 9, 10, 11, 12, 13 and 14

A great deal of research has focused on the empirical identification of the network structure from microarray or other genomic data. An established method called genome-wide location analysis, which uses chromatin immunoprecipitation with epitope-tagged transcription factors followed by hybridization to DNA microarrays spotted with intergenic sequences (ChIP-chip), has determined the relationships between transcription factors and the target genes they regulate on a global scale in budding yeast (Lee et al. 2002; Harbison et al. 2004). Starting with the network of 106 transcription factors identified by Lee et al. (2002), we considered only those transcription factors that had been previously annotated as involved in the ESR. We also considered the transcription factors that regulated them and those that they regulated, regardless of annotation. The confidence level of these regulatory relationships reported by Lee et al. (2002) was $p<0.001$. The largest connected cluster of nodes became the gene regulatory network, comprised of 21 nodes and 31 edges. All of the edges were confirmed with the data from a second genome-wide location dataset from Harbison et al. (2004). The standard names for the transcription factors in the gene regulatory network are listed in “Appendix”, along with their systematic names and aliases from the *Saccharomyces* Genome Database (http://www.yeastgenome.org), and the network structure itself is pictured in Fig. 1. Each node simultaneously represents the gene, the mRNA, and the protein. For the sake of simplicity, in the rest of the paper, we will refer to the nodes as “genes” even though the node represents all three entities. Each directed edge represents the regulatory relationship between two nodes. This means that the transcription factor encoded by the gene at the originating node either activates or represses expression of the gene at the recipient or target node. We emphasize that the arrows do not denote activation here; rather, we are indicating the directionality of regulation.

**Fig. 2:**
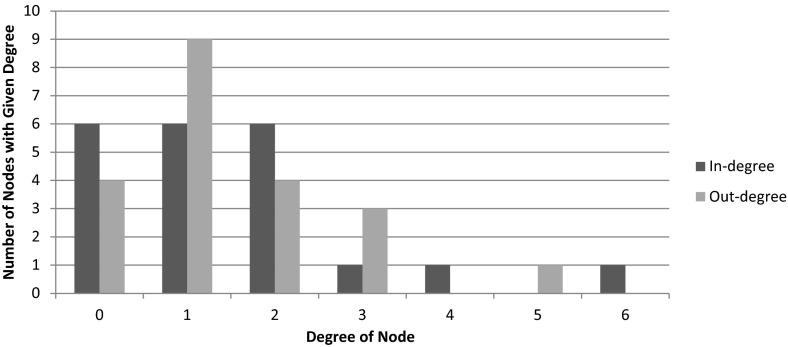
In-degree (*dark*) and out-degree (*light*) distribution of directed edges in the gene regulatory network

This graph contains a total of 21 nodes and 31 edges. Of the 21 nodes, 15 are regulated by at least one gene in the network. The in-degree and out-degree distributions of the nodes are given in Fig. 2.

One observation from this histogram is that 6 nodes have in-degree 0, meaning that those 6 nodes are not controlled by any of the genes in the network. Furthermore, four of the nodes have out-degree 0, meaning that they do not control any of the genes in the network. One gene, RAP1, has out-degree 5, making it influential to the most genes. The gene YAP6 is influenced by 6 genes. Four genes show autoregulation: AFT1, NRG1, RAP1, and YAP6. The deepest regulatory chain includes 5 nodes (originating at SKN7), with 4-node chains originating at CIN5, MAC1, PHD1, SKN7, and YAP1. Most nodes have a single input or are part of a simple regulatory chain, but several participate in complex feedforward motifs (CIN5, ROX1, and YAP6; SKN7, YAP1, and ROX1). Furthermore, there appears to be two distinct subnetworks (upper left and lower right of Fig. 1) that are only connected through edges originating at ABF1 and PHD1. This complexity of network structure makes it difficult to hypothesize up front what the regulatory dynamics might be and necessitates use of a model to explicate them.

After defining the network topology, the next step in the modeling process is the determination of the dynamics, including the signs (activation/repression) and the influence magnitudes of the regulatory relationships. However, we first describe in more detail the nature of the microarray data that we will use to infer parameters in the model.

## Cold Shock DNA Microarray Data

We are grateful to Babette Schade for providing the complete microarray dataset for wild type yeast subjected to cold shock as published in Schade et al. (2004). In their experiment, wild type *Saccharomyces cerevisiae* strain BY4743 grown at $30\,^{\circ }\hbox {C}$ in rich YEPD medium was shifted to $10\,^{\circ }\hbox {C}$. Samples were collected before cold shock $(t_{0})$, and after $10 (t_{10})$, $30 (t_{30})$, $120 (t_{120})$ minutes, and 12 and 60 h of cold shock. We restricted our analysis to the first three cold shock timepoints because we are specifically interested in the early response to cold temperatures in yeast. As discussed in Sect. 2, there are substantial biological differences between the early and late cold shock responses which would lead to substantial differences in the dynamics of the early response which occurs on the timescale of minutes to hours and the late response which occurs on the timescale of hours to days. The dataset we obtained had three replicates for the $t_{0}$ timepoint, seven replicates of the $t_{10}$ timepoint, six replicates of the $t_{30}$ timepoint, and four replicates of the $t_{120}$ timepoint. We assumed that each replicate of the $t_{0}$ timepoint consisted of a competitive hybridization of Cy3-labeled cDNA derived from one culture grown at $30\,^{\circ }\hbox {C}$ with Cy5-labeled cDNA derived from a different culture grown at $30\,^{\circ }\hbox {C}$. We also assumed that the replicates of the $t_{10}$, $t_{30}$, and $t_{120}$ timepoints consisted of competitive hybridizations of labeled cDNA from independently cold shocked cultures to labeled cDNA from control cultures grown at $30\,^{\circ }\hbox {C}$. The data we obtained had already been subjected to within-chip normalization. We performed the following manipulations on the data. The expression ratios (fold changes) were $\hbox {log}_{2}$ transformed. Between-chip normalization was carried out (see Stekel 2003 for a detailed discussion of microarray normalization). Each replicated measurement of $\hbox {log}_{2}$ ratio (that is, each individual microarray chip) was mean removed and scaled by subtracting the average $\hbox {log}_{2}$ ratio for all of the spots on the microarray from each spot and dividing each spot by the standard deviation of all spots on the microarray. For each gene at each timepoint we computed the average $\hbox {log}_{2}$ ratio of the replicate measurements to produce one data point, along with the standard deviation. We also computed a modified *t* statistic to determine whether each average $\hbox {log}_{2}$ ratio was significantly different than zero and a *p* value based on the *t* statistic. We should note that the variability and the small number of replicates make for tests that are not very powerful. Table 1 shows the number and percentage of genes in the dataset with significant changes in gene expression at three different *p* value cut-offs, $p<0.05$, $p<0.01$, and $p<0.001$. The $t_{0}$ timepoint has very few genes with significant changes in expression as would be expected when labeled cDNA from two control cultures are hybridized against each other. However, the fact that 2.6 % of the genes did actually meet the $p<0.05$ criterion for significant differential expression points to the variability, both technical and biological, in this experimental system. The other timepoints all have a greater number of genes showing a significant change in expression than would be expected by chance using that particular *p* value cut-off, except for the $t_{30}$ timepoint at $p<0.001$. This demonstrates that the yeast did indeed respond to the cold shock treatment at $10\,^{\circ }\hbox {C}$ with changes in gene expression.

**Table 1:** Number and percentage of genes with significant changes in gene expression at each timepoint for three different *p* value cut-offs

Timepoint	*p* value cut-off		
	$p<0.05$	$p<0.01$	$p<0.001$
$t_{0}$	170 (2.6 %)	31 (0.48 %)	1 (0.015 %)
$t_{10}$	822 (12.8 %)	294 (4.6 %)	72 (1.1 %)
$t_{30}$	785 (12.2 %)	251 (3.9 %)	42 (0.07 %)
$t_{120}$	1361 (21.2 %)	522 (8.1 %)	111 (1.7 %)

In Table 2, we provide the average $\hbox {log}_{2}$ ratios and *p* values for the 21 genes in our network. Notably, only nine genes in the network show significant changes in gene expression at $p<0.05$ at any timepoint. ABF1, FHL1, and HSF1 show significant decreases in gene expression at one or more cold shock timepoints, and MAC1, MSN4, RAP1, and RPH1 show significant increases in gene expression at one or more cold shock timepoints. AFT1 and ROX1 have $p<0.05$ for decreases in expression observed at the $t_{0}$ timepoint, when no change in expression is expected.

**Table 2:** Average $\hbox {log}_{2}$ ratios of expression and *p* values derived from Schade et al. (2004)

Gene	$t_{0}$		$t_{10}$		$t_{30}$		$t_{120}$	
	Average $\hbox {log}_{2}$ ratio	*p* value	Average $\hbox {log}_{2}$ ratio	*p* value	Average $\hbox {log}_{2}$ ratio	*p* value	Average $\hbox {log}_{2}$ ratio	*p* value
ABF1	1.6210	0.4101	$-$0.3537	0.0155	$-$0.2690	0.2631	$-$1.2538	0.0205
ACE2	$-$0.5424	0.2899	$-$0.0248	0.9103	$-$0.4154	0.4755	$-$0.3487	0.6256
AFT1	$-$0.3285	0.0313	0.3965	0.0718	0.1158	0.7717	0.0584	0.8614
CIN5	$-$0.2350	0.7514	$-$0.0741	0.7375	$-$0.0457	0.7625	0.4844	0.2610
CUP9	0.4326	0.3202	$-$0.0307	0.8705	$-$0.1631	0.4870	$-$0.8179	0.0842
FHL1	$-$0.5464	0.2285	$-$0.1777	0.1812	$-$0.2368	0.2198	$-$0.7515	0.0125
GTS1	$-$0.3374	0.4561	$-$0.1894	0.4621	0.1224	0.4558	0.8562	0.0732
HAL9	0.1967	0.6944	$-$0.2153	0.3542	0.0859	0.2757	$-$0.3585	0.4513
HSF1	$-$0.0039	0.9900	$-$0.1460	0.0216	$-$0.7799	0.0270	$-$0.3743	0.1788
MAC1	$-$0.7799	0.1106	$-$0.1774	0.4047	0.0761	0.8014	0.5849	0.0285
MSN1	$-$0.1416	0.6824	$-$0.4139	0.1028	0.0893	0.7184	0.0470	0.1496
MSN4	$-$0.0071	0.9877	0.2969	0.0662	0.2576	0.4856	1.1248	0.0201
NRG1	$-$0.4413	0.5057	$-$0.1239	0.6252	0.5153	0.3895	$-$0.3026	0.5371
PHD1	$-$0.0206	0.9677	0.3247	0.3541	0.5707	0.1099	0.1076	0.2342
RAP1	$-$0.2247	0.5158	$-$0.0227	0.9208	0.3397	0.5221	0.5514	0.0417
REB1	0.0752	0.9011	0.1992	0.4729	0.2667	0.2346	0.3491	0.3006
ROX1	$-$0.3507	0.0194	$-$0.2929	0.1053	0.2343	0.3230	$-$0.2117	0.5370
RPH1	0.6766	0.0613	1.1363	0.0021	0.8952	0.0148	0.7032	0.0049
SKN7	0.1884	0.8444	0.0355	0.7730	0.1685	0.6378	0.9352	0.1036
YAP1	$-$0.6525	0.6474	0.1897	0.5041	0.3097	0.3116	1.3499	0.0888
YAP6	0.1345	0.7037	$-$0.2543	0.7110	0.0780	0.7583	0.2820	0.1740

## Mathematical Modeling of Regulatory Networks

Gene regulation can be modeled with a wide variety of mathematical structures at many levels of resolution. Schlitt and Brazma (2007) review four levels at which gene regulatory networks have been modeled: (1) parts lists, (2) topology models, (3) control logics models, and (4) dynamic models. Karlebach and Shamir (2008) provide a similar breakdown of gene regulatory modeling, into logical models, continuous models, and single-molecule models. In many cases, trade-offs between the number of genes included in the model and the level of detail of the model govern the modeling structure that is chosen and applied. Parts lists and topology models concern themselves with the identity and connectivity of genes in the model on the scale of the entire genome, transcriptome, or proteome, while kinetic models often focus on small systems where detailed experimental data are available (e.g., the $O_\mathrm{R}$ control system of bacteriophage lambda, Shea and Ackers 1985). In the case of the early cold shock response, we want to scale down from the whole-genome topology model to more closely investigate a smaller gene regulatory network. Because a master regulator for this response, akin to HSF1 for heat shock, has not been identified for cold shock, our network must still be large enough to include all potential regulators annotated as being involved in the ESR. And because we want to discover the relative influence of this set of factors and their activation/repression relationships, we want to investigate the dynamics of the network. In short, to understand the cell’s early response to cold shock, we must combine topology and dynamic models on a medium scale in a way that has predictive power to understand the interactions in gene regulatory networks.

Taking a step in that direction, we build a model of gene regulation that adds the dynamics of transcription factor production onto their interaction network. Research along these lines has applied differential equation structures (e.g., Alon 2007; Wilkinson 2006; Vohradský 2001; Vu and Vohradsky 2007; Kauffman et al. 2003; Climescu-Haulica and Quirk 2007; Chen et al. 2005, 1999; Blossey et al. 2008), typically treating the problem as one of mass balance.

The basic balance concept is one of production and degradation. The equation

1 $$\begin{aligned} \dot{x}_i (t)=p_i (x(t))-d_i x_i (t) \end{aligned}$$

in which the function $p_i$ gives the production rate, and the linear term $d_i x_i (t)$ is the degradation rate, defines an in-flow, out-flow conservation principle for the level of expression $x_i (t)$ over time. The functions $p_{i}$ will of course depend on expression levels of all the genes controlling gene *i*. Commonly used structures for the production functions include linear (Chen et al. 1999), quadratic (Angeli et al. 2009; Sontag 2007), Michaelis-Menten (Alon 2007; Cao and Zhao 2008), and sigmoidal (Chen et al. 2005; Mendoza and Xenarios 2006; Smolen et al. 2000; Vu and Vohradsky 2007). The form of $p_i$ is thus a primary modeling issue.

The production function that we adopt here, based on a sigmoidal production model proposed in Vu and Vohradsky (2007), takes the general form

2 $$\begin{aligned} p_i (x(t),\theta )=\frac{P_i }{1+\exp \left( {-\sum \limits _j {w_{ij} (x_j (t)-\tau _{ij} )} } \right) } \end{aligned}$$

in which $P_i$ is the maximal rate of expression (i.e., the production rate at full production activation), $w_{ij}$ is the interaction weight of gene *j* in regulating gene *i*, and $\tau _{ij}$ is a threshold expression level at which production switches “on” and “off.” In this functional form, the parameter $\theta$ captures the weights, thresholds, and possibly even the baseline production rates.

We first note that the interaction network is contained in the weight parameters. If the weight $$w_{ij} $$ is nonzero, then an edge connects the production of gene or node *i* with the expression level $$x_j $$. For example, the graph of Fig. 1 has 31 edges. We emphasize that the network is a directed graph: the expression of transcription factor *j* may affect that of *i* without the converse relationship necessarily holding. We also note that the sign of the weight governs the type of relationship: positive weights correspond to activation, while negative weights correspond to repression.

**Fig. 3:**
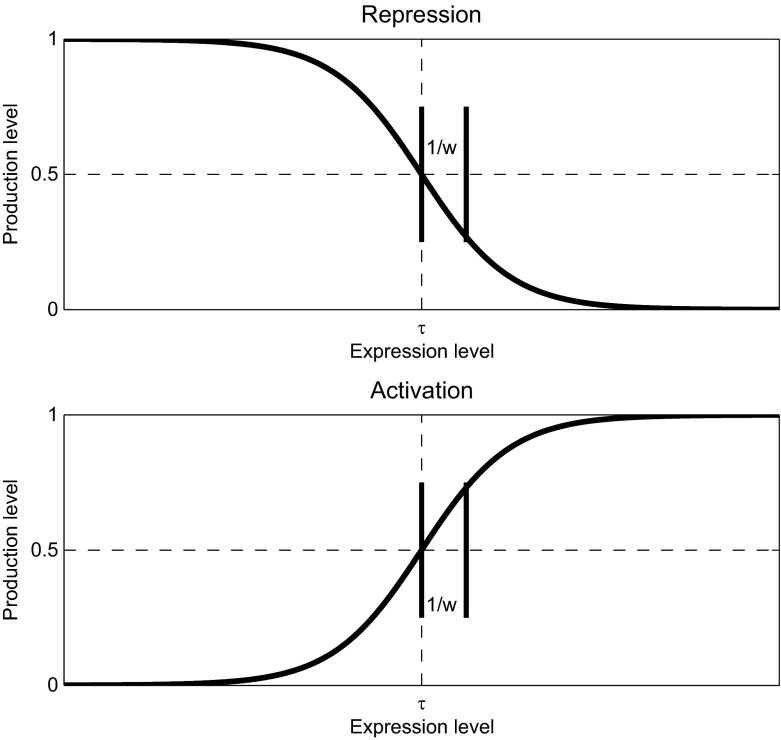
Sigmoidal repression (*top*) and activation (*bottom*) functions

The functional form of the sigmoid $S(u;w,\tau )=1/(1+e^{-w(u-\tau )})$ on which Eq. (2) is based is more easily understood with a graph. In Fig. 3, we show the basic shape of repression and activation production functions of the form $S(w(u-\tau ))$ versus *u*.

Roughly speaking, we think of production as turning on and off, depending on the expression levels of activating and repressing transcription factors. The weight governs the “boundary layer” between on and off states, and the threshold governs the input level at which the switch is thrown. For very large weights, the production function approximates the unit step or Heaviside function with jump positioned at the threshold value. For an activator, expression levels above the threshold lead to production, while expression levels below turn production off. Likewise, repressors turn production off at higher-than-threshold levels and turn production on when expression levels decrease below the threshold.

Generally speaking, the transient behavior of the system (1) must be determined numerically. Long-time behavior issues, such as equilibria and their stability, are quite difficult for systems of the size under study here: the specific example of cold shock in yeast we discuss below involves 21 state variables. Our interest in this paper is in the determination of parameters from data, so we do not undertake any analysis of long-time behavior, other than to note that the work of Angeli et al. (2009) provides an interesting approach to stability through the notion of a coherent system.

With a model of dynamic regulation in hand, we now turn to the determination of parameter values for the model. The system of differential equations we have presented in (1) is a complex model with a large number of parameters. When considered in the context of fitting this model to microarray data, which is expensive and time consuming to collect, we must take great care in our parameter estimation procedures. Here we discuss a number of issues associated with parametric dependence and parameter estimation.

As discussed in Sect. 3, the microarray data we use provides a measurement of the level of gene expression activity at the time of measurement relative to the initial expression at $$t_{0}$$. We denote by $$\hat{{x}}_i^r (t_k )$$ the *r*th replicate observation of gene *i* expression level at time $$t_k $$. The parameter identification process then becomes a problem of comparing the model form

$$\begin{aligned} \dot{x}_i (t;\theta )=\frac{P_i }{1+\exp \left( {-\sum \limits _j {w_{ij} (x_j (t;\theta )-\tau _{ij} )} } \right) }-d_i x_i (t;\theta ) \end{aligned}$$

to the observed data. Here we have explicitly included the dependence of the state variable on the rate and network parameters, which comprise the vector $\theta$.

The most common approach to the estimation of parameters for models such as our gene regulatory network model is that of least squares. A form of nonlinear regression, the least square approach compares model output to observed data and chooses the parameter estimate by minimizing this discrepancy. In particular, the function

$$\begin{aligned} J(\theta )=\sum _{s=1}^R {\sum _{k=1}^{N_T } {\sum _{i=1}^{N_G } {\left| {\log _2 \hat{{x}}_i^s (t_k )-\log _2 x_i (t_k ,\theta )} \right| ^{2}} } } \end{aligned}$$

is to be minimized, in which $\hat{{x}}$ denotes observed expression levels from the microarray data, and $x(t,\theta )$ denotes the parameter dependent solution of the differential equation. Here we are assuming *R* repetitions of the experiment, which is observed at times $t_k ,k=1,2,\ldots ,N_T$ for all genes in the network $(i=1,2,\ldots ,N_G )$. We also note the use of the $\hbox {log}_{2}$ transform, which as noted in Sect. 3 is commonly applied to microarray data.

This type of estimation problem has been studied by a number of investigators, including the definitive text (Gallant 1987), the papers (Banks and Fitzpatrick 1990; Fitzpatrick 2008) and the monograph (Huet et al. 2004).

We note that the model requires potentially a very large number of parameters. In the “worst” case, if the regulatory network forms a connected graph with *n* nodes, then there are $n^{2}$ weights and $n^{2}$ thresholds. While the number of parameters is a serious concern, the difficulty in identifying the thresholds is perhaps the most significant problem. Note that

$$\begin{aligned} \sum _j {w_{ij} (x_j (t)-\tau _{ij} )} =\sum _j {w_{ij} x_j (t)} -\sum _j {w_{ij} \tau _{ij} =} \sum _j {w_{ij} x_j (t)} -b_i , \end{aligned}$$

where

$$\begin{aligned} b_i =\sum _j {w_{ij} \tau _{ij} } \end{aligned}$$

defines a new parameter, $b_{i}$. We note that, for any choice of weights with at least two being non-zero, there are an infinite selection of thresholds that would produce identical model dynamics, making the thresholds non-identifiable. Thus, for the purposes of parameter identification, we reduce the thresholds down to the *b* parameters. This parameterization was also used by Vu and Vohradsky (2007). While the individual threshold parameterization holds a slightly more intuitive meaning, in terms of the expression level in each controller gene that “turns the switch,” the *b* parameter represents a “net threshold” at which the combined level of activities leads to switching.

We thus denote by $\theta$ the parameter vector $\theta =(w,b,P),$ in which the number of individual *w*’s is governed by the total number of edges in the network, the number of *b*’s is governed by the sum of the in-degrees of each node, and the number of *P*’s is governed by the number of nodes. As noted in Sect. 2, our network involves 31 weights, 15 *b*’s, and 21 production rates.

We denote by $\hat{{\theta }}$ the minimizer of the least squares cost. Generally speaking, one must determine this minimizer numerically with an iterative optimization procedure. Some theoretical results pertaining to the estimator, however, are available. For example, statistical results from the references above pertain to modeling the observations. If we assume that

$$\begin{aligned} \hat{{x}}_i^s (t_k )=x_i (t_k ,\theta ^{{*}})+\varepsilon _{ik}^s , \end{aligned}$$

where the errors $\varepsilon _{ik}^s$ are zero mean, finite variance, independent and identically distributed random variables, then parameter estimator obeys a central limit theorem:

$$\begin{aligned} \sqrt{n}\left( {\hat{{\theta }}-\theta ^{{*}}} \right) \sim N(0,\Sigma ), \end{aligned}$$

as $n\rightarrow \infty$, where $\Sigma =\sigma ^{2}V^{-1}$ and $\sigma ^{2}$ is the noise variance in the observations. The matrix *V* is the sensitivity matrix, given by

$$\begin{aligned} V=\sum _i { \int \limits _{t_0 }^{t_f } {\frac{\partial x_i }{\partial \theta }(t,\theta ^{{*}})\frac{\partial x_i }{\partial \theta }^{{T}}(t,\theta ^{{*}})\mathrm{d}t} } \end{aligned}$$

in which $\frac{\partial x_i }{\partial \theta }$ denotes the gradient of gene *i* expression levels with respect to the parameter vector and with the superscript *T* as its transpose. The asymptotic as stated involves in-fill sampling in time, but other types of asymptotics are available (see, e.g., Banks and Fitzpatrick 1990; Fitzpatrick 2008; Gallant 1987). This matrix is related not only to the covariance of the parameter estimator but also to the numerical conditioning of the optimization procedure.

A more complex and robust approach to parameter estimation is Bayesian estimation. In Bayesian statistical inference, one begins with a prior distribution, $\pi$. This distribution quantifies our a priori information concerning the parameters. The second component of the Bayesian approach is the conditional distribution of the measurement, given the parameter, $p(x|\theta )$. Inference (e.g., estimation, hypothesis testing) is performed through the posterior distribution, computed via Bayes’ formula:

$$\begin{aligned} \pi (\theta |x)=\frac{p(x|\theta )\pi (\theta )}{\int \limits _\Theta {p(x|\theta ^{\prime })\pi (\theta ^{\prime })\mathrm{d}\theta ^{\prime }} },\quad \theta \in \Theta . \end{aligned}$$

An interpretation of Bayesian analysis that is particularly appealing in applications is that the prior and posterior represent quantifications of our uncertainty in parameter values before and after experimental data has been collected. A full coverage of Bayesian analysis, including philosophy, conceptual structure, analysis, and application, is contained in the excellent text of Berger (1993).

Bayesian maximum likelihood, in which one determines the parameter estimator by maximizing the posterior density, corresponds to a type of penalized least squares. If we assume, for example, that the errors $\varepsilon _{ik}^s$ are zero mean normally distributed random variables and that the prior is of an exponential family, $\pi (\theta )=C\exp \left( {-G(\theta )} \right)$, then the negative of the log of the posterior is

$$\begin{aligned} -\ln \left( {\pi (\theta |y)} \right)= & {} \frac{\sum \limits _{s=1}^S {\sum \limits _{k=1}^{N_T } {\sum \limits _{i=1}^{N_G } {\left| {\log _2 \hat{{x}}_i^s (t_k )-\log _2 x_i (t_k ,\theta )} \right| ^{2}} } } }{2\sigma ^{2}}+G(\theta )-\ln (C)\nonumber \\&+\,\frac{1}{2}\ln (2\pi \sigma ^{2}). \end{aligned}$$

The last two terms in this expression are independent of the parameter and thus irrelevant to parameter estimation. We may then take as our penalized least squares criterion

$$\begin{aligned} \tilde{J}(\theta )=\sum _{s=1}^S {\sum _{k=1}^{N_T } {\sum _{i=1}^{N_G } {\left| {\log _2 \hat{{x}}_i^s (t_k )-\log _2 x_i (t_k ,\theta )} \right| ^{2}} } } +G(\theta ) \end{aligned}$$

with the function *G* representing our prior level of uncertainty in the parameter’s value. The form of *G* is often taken to be a quadratic, an assumption equivalent to using a normal prior. This approach to estimation is also called penalized least squares. In this work, we use a quadratic *G* with a scaling factor $\alpha$ to control the relative role of data noise and parameter sensitivity (where $\theta _{0}$ denotes our best a priori estimate, as well as the prior mean):

$$\begin{aligned} \tilde{J}_\alpha (\theta )=\sum _{s=1}^S {\sum _{k=1}^{N_T } {\sum _{i=1}^{N_G } {\left| {\log _2 \hat{{x}}_i^s (t_k )-\log _2 x_i (t_k ,\theta )} \right| ^{2}} } } +\alpha \left| {\theta -\theta _0 } \right| ^{2}. \end{aligned}$$

**Table 3:** Degradation rates for transcription factor proteins

Gene	Degradation rate
ABF1	0.3466
ACE2	0.2310
AFT1	0.0301
CIN5	0.0272$^\mathrm{a}$
CUP9	0.0257
FHL1	0.0173
GTS1	0.0110
HAL9	0.0272$^\mathrm{a}$
HSF1	0.0272$^\mathrm{a}$
MAC1	0.0075
MSN1	0.0770
MSN4	0.0272$^\mathrm{a}$
NRG1	0.0693
PHD1	0.0495
RAP1	0.0165
REB1	0.0578
ROX1	0.0133
RPH1	0.0126
SKN7	0.0301
YAP1	0.0301
YAP6	0.0330

$^\mathrm{a}$ Genes for which a median degradation rate was used for missing values from Belle et al. (2006) (CIN5, HSF1, MSN4, HAL9)

The choice of the parameter $\alpha$ can be challenging, and there are many approaches to its selection, including cross validation (Golub et al. 1979) and the L-curve (Hansen and O’Leary 1993), the technique we examine here. The L-curve method involves the computation of a parametric plot of the least squares residual versus the penalty term, parameterized by $\alpha$. For each $\alpha$, we compute the minimizer $\hat{{\theta }}_\alpha$of $\tilde{J}_\alpha$, and then we compute $\tilde{J}_0 (\hat{{\theta }}_\alpha )$ (the least squares residual error) and $r(\hat{{\theta }}_\alpha )=\left| {\hat{{\theta }}_\alpha } \right| ^{2}$ (the penalty). In this procedure, we plot $r(\hat{{\theta }}_\alpha )$ versus $\tilde{J}_0 (\hat{{\theta }}_\alpha )$ for each $\alpha$. Typically, this plot takes the shape of an L, the corner of which is used to select an appropriate penalty level. The additional computation required to perform the L-curve analysis pays significant dividends in practice. Working from larger values of $\alpha$ to smaller ones aids in the numerical optimization, as the output of the more highly penalized optimization provides an improved starting point for the less penalized one to follow.

In Sect. 5 below, we illustrate the penalized least squares and L-curve technique with microarray data as published in Schade et al. (2004). Having reviewed the basic concepts of dynamic modeling and parameter estimation, we turn to the specific problem of interest, inferring the regulatory dynamics of the early response to cold shock in *S. cerevisiae*.

## Issues of Parameter Estimation and Model Sensitivity

In considering the particular aspects of our 21-state model, we see that there are 21 production rate parameters, 21 degradation rate parameters, 31 weights, and 15 net thresholds. Such a large number of parameters brings about a major challenge within the context of the microarray data we are using, in which we have 3–7 replicates reporting $\hbox {log}_{2}$ fold changes in expression for each gene at 4 time points.

First, we will assume that the degradation rates are known or obtainable through other means. To find the degradation rate, we used published protein half-life data from Belle et al. (2006). We converted the half-life data values to the degradation rates by taking the natural log of the half-life and dividing by 2 (Table 3). For several transcription factors, the half-life data were not available, so we computed a median of the half-life values for the other transcription factors, converted it and used that value for those proteins. The median was based on the half-lives reported by Belle et al. (2006) for 142 proteins for which there were data out of 203 proteins annotated as transcription factors by Harbison et al. (2004).

The data we obtain from microarrays are in the form of expression relative to time 0 expression, $$x_i (t)=\textit{mRNA}_i (t)/\textit{mRNA}_i (0)$$, leading to theoretical initial values of 1 for all expression levels in the dynamics. In all model simulations, we specify $$x_i (0)=1$$ for all genes. Moreover, were the system not cold shocked, we would expect it to be in equilibrium at constant (relative) expression of 1 with no transcriptional regulation occurring, i.e., $$\sum \limits _j {w_{ij} -b_i } =0$$. Thus, we would expect the non-cold-shocked system to have threshold values for $$x_{i}(t)$$ equal to one, leading to the steady-state equations of $$\frac{P_i }{1+\exp (0)}-d_{i}\, ^{*}1=0$$, or $$P_i =2d_i $$.

We do not use this approach to estimate production rates for the following reason: several of the equations, associated with genes not receiving activation or repression signals from within the network, are independent of the parameter estimation process. Thus, these genes would be in steady state, and we could then drop them from the dynamical system and estimation. We do find that this estimation approach does give us a reasonable initial guess for any iterative optimization algorithm we apply to minimize the penalized least squares cost. We emphasize that this produces an initial guess for production rate parameters; it is not an initial condition for the dynamical system, nor are any cold shock dynamics assumed or forced to be in steady state.

The data we use for the penalized least squares estimation come from the experiments reported in Schade et al. (2004; see Sect. 3 and Table 2).

**Fig. 4:**
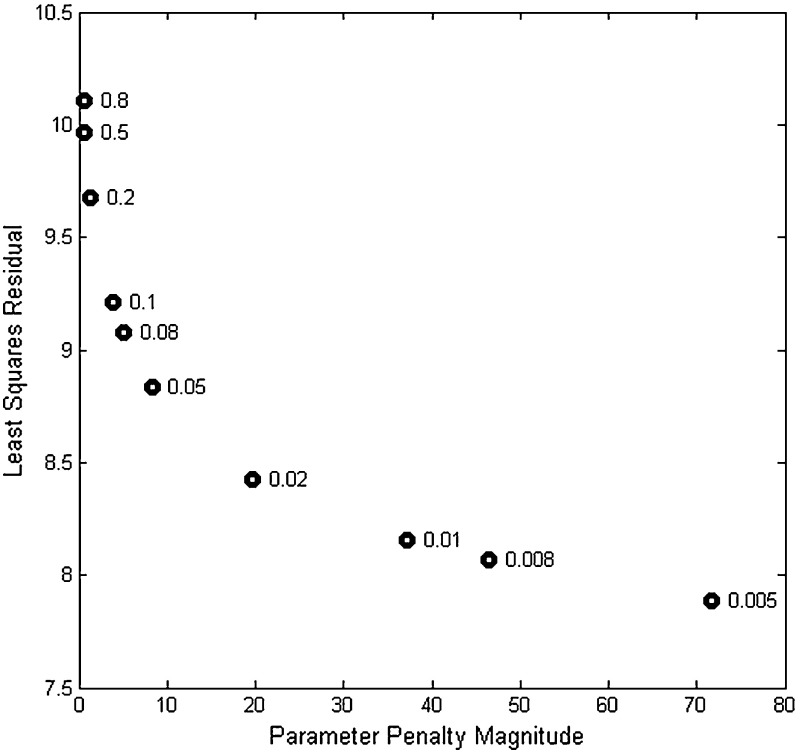
L-curve analysis of Schade et al. (2004) data as fit to model. Values of $\alpha$ annotate the points

The least squares criterion takes the form

$$\begin{aligned} \tilde{J}_\alpha (\theta )=\sum _{k=1}^4 {\sum _{i=1}^{15} {\left| {\log _2 \hat{{x}}_i (t_k )-\log _2 x_i (t_k ,\theta )} \right| ^{2}} } +\alpha \left| w \right| ^{2}+\alpha \left| b \right| ^{2}+\alpha \left| P \right| ^{2}, \end{aligned}$$

in which we apply the L-curve method to determine an appropriate value for $\alpha$. Our numerical implementation in MATLAB (Release R2010a) uses the optimization toolbox routine fmincon to perform the minimization. We use a constrained minimization algorithm to maintain non-negative production rates. In producing this L-curve, we start with a fairly large value of $\alpha$, so that the minimization is dominated by the penalty. Initial guesses for the weights are all set to 1, and initial guesses for the net thresholds are set to 0. The production rates are initialized as discussed above. Once the minimization iteration has reached numerical convergence, the resulting optimal parameters are used to initialize the minimization for the next smaller penalty parameter. In Fig. 4, we provide the L-curve obtained through this procedure.

**Fig. 5:**
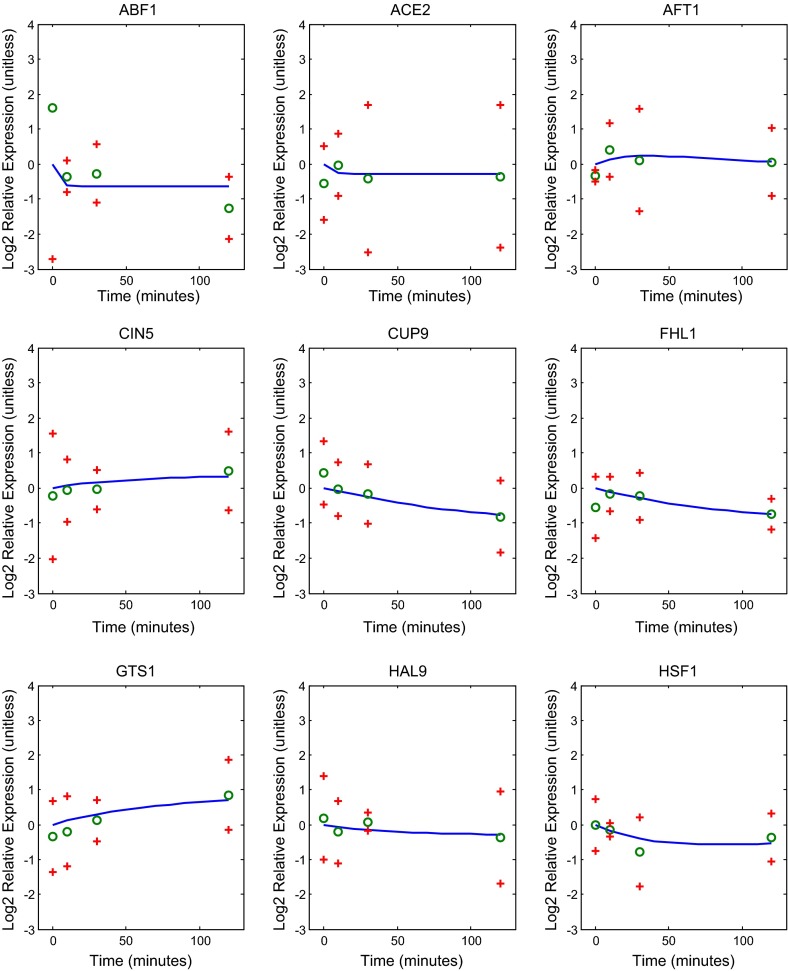
Genes ABF1, ACE1, AFT1, CIN5, CUP9, FHL1, GTS1, HAL9, HSF1 in the regulatory network: best fit model dynamics and data. Relative expression level is plotted as $\hbox {Log}_{2}$ fold change (ratio) over time. The *solid blue curve* in each panel gives the model with the best fit parameters. The *green circles* represent the data, and the *red crosses* provide a 95 % confidence interval for the data. The *upper point* of the confidence interval for ABF1 at $t_{0}$ extends outside of the graphic coordinate limits

**Fig. 6:**
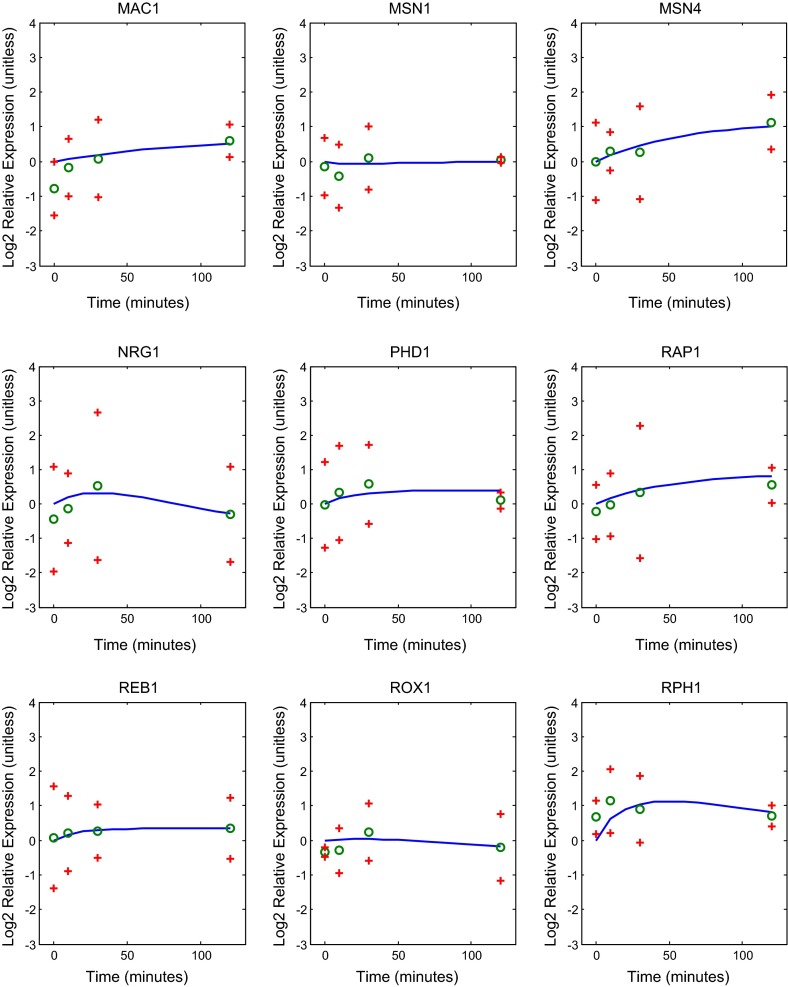
Genes MAC1, MSN1, MSN4, NRG1, PHD1, RAP1, REB1, ROX1, RPH1 in the regulatory network: best fit model dynamics and data. Relative expression level is plotted as $\hbox {Log}_{2}$ fold change (ratio) over time. The *solid blue curve* in *each panel* gives the model with the best fit parameters. The *green circles* represent the data, and the *red crosses* provide a 95 % confidence interval for the data

**Fig. 7:**
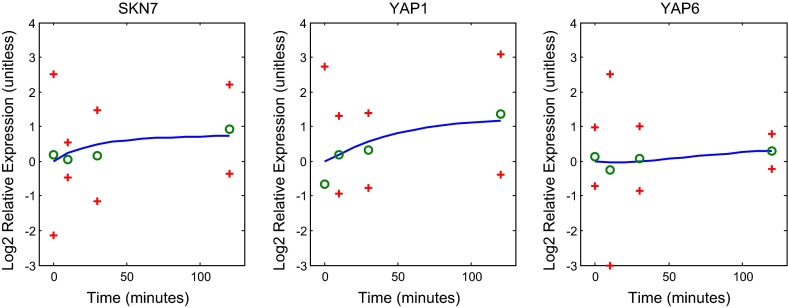
Genes SKN7, YAP1, and YAP6 in the regulatory network: best fit model dynamics and data. Relative expression level is plotted as $\hbox {Log}_{2}$ fold change (ratio) over time. The *solid blue curve* in *each panel* gives the model with the best fit parameters. The *green circles* represent the data, and the *red crosses* provide a 95 % confidence interval for the data

**Fig. 8:**
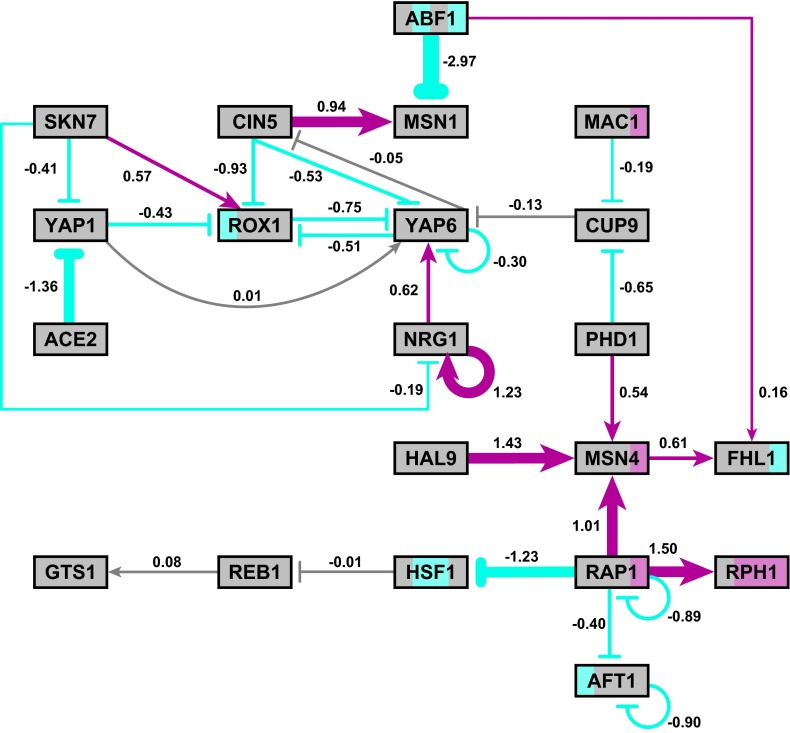
Weights and experimental expression data displayed on the network diagram. The sign of the weight (positive for activation and negative for repression) is represented by both the arrowhead type (pointed or blunt, respectively) and edge color (*magenta* and *cyan*, respectively, or *gray* for weights near zero). The magnitude of the weight is represented by the *thickness of the edge*; larger weights are represented by *thicker lines*. The weight value is noted next to *each edge*. *Each node* is colored based on the Schade et al. (2004) expression data. There are four stripes for the four timepoints, $t_{0}$, $t_{10}$, $t_{30}$, $t_{120}$. The stripe is *gray* if there was no significant change in expression at that timepoint, *magenta* if there was a significant increase in expression, and *cyan* if there was a significant decrease in expression $(p<0.05)$. An interactive version of this diagram can be viewed online at http://dondi.github.io/GRNsight/index.html

**Fig. 9:**
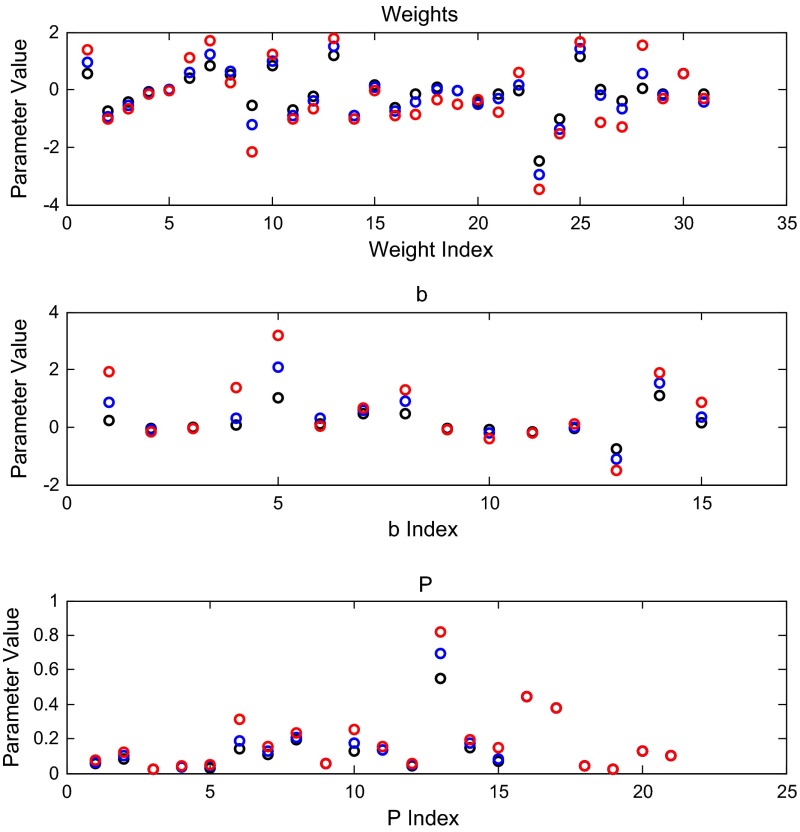
Weight (*top*), *b* (*middle*), and production rate (*bottom*) parameter comparisons for $$\alpha =0.02$$ (*black*), 0.01 (*blue*), and 0.005 (*red*) from penalized least squares estimation. Weight, $$w_{ij} $$, (*top panel*), net threshold, *b* $$_{i}$$, (*middle panel*), and production rate, $$P_i $$, (*bottom panel*)

The L-curve suggests three possible good $\alpha$ values to select. In Fig. 9 we compare the weight, net threshold, and production parameter values for $\alpha = 0.02$, 0.01, and 0.005. We selected the value $\alpha =0.01$ for the remainder of the analyses presented below. In Figs. 5, 6 and 7, we show the dynamics of each gene’s expression. The solid blue curve in each panel gives the model with the best fit parameters. The green circles represent the data, and the red crosses provide a 95 % confidence interval for the data. Genes without significant changes in expression (Table 2; Fig. 8) show little change in dynamics over time.

The parameter estimates derived from the minimization are given in Table 4. The electronic supplementary material is a zipped file containing the corresponding input spreadsheet and output spreadsheet. The MATLAB code is available upon request.

Figure 8 shows the weights and experimental expression data displayed on the network diagram.

We conducted a number of additional computations to explore the quality of these estimates. First, we compared the estimated parameter values for several of the L-curve runs. In Fig. 9, we plot the weights, net thresholds (*b*’s), and production rates from three different penalty levels.

We see that the magnitudes of the parameters are different, but that the trends and patterns agree for all $\alpha$ values in the penalized least squares estimation. The signs of the weight and thresholds, in particular, stay the same, and the production rates for a number of the genes are quite close. The parameter index is used for simplicity of plotting: Table 5 connects the indexing with the genes for Fig. 9 and subsequent figures (weight indexes are annotated on the edges of Fig. 1).

In a second test, we randomized the initial guesses for the iterative optimization scheme. We ran the minimization routine using 10 different initial guesses for each individual parameter. In the cases of the weights and thresholds, we sampled from a standard normal distribution, and for the production rates (which must be nonnegative), we multiplied the optimal production rates by a normal with mean 1 and standard deviation 0.03, truncating to 0 if negative. Using the penalty parameter $\alpha =0.01$, we found that the resulting optimal parameter values were quite stable. In Tables 6, 7, and 8, we provide the standard deviations of the randomly selected initial guesses from the ten individual computations as well as the standard deviations of the resulting estimated parameters.

**Fig. 10:**
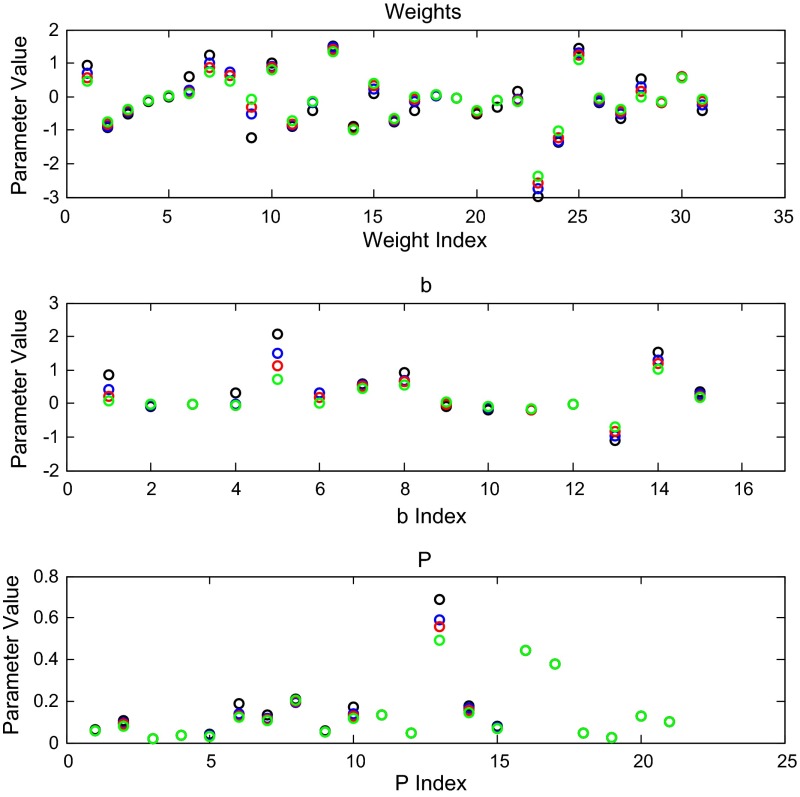
Estimated weights from three different model-generated data fits. *Black circles* denote the parameters used to generate the data. *Blue circles* denote the estimated parameters from 5 min time step data; *red* 10 min time steps; *green* 20 min time steps

As a final test of the estimation routine’s accuracy, we performed some tests using model-generated data. We used the parameters in Table 4 to simulate data by solving the differential equation system (1). From the simulation, we used model-generated data in 5, 10, and 20 min time steps to conduct the penalized least squares fit, again with $\alpha =0.01$. Figure 10 contains the resulting parameter estimates.

Since we have no *a priori* knowledge concerning the quality of the model or the parameter values, we cannot say with certainty that our fit, as detailed in Figs. 5, 6, and 7, and Table 4, are “correct” or even “close to the truth.” The additional tests of randomized initial guesses and model-generated data lend confidence, however, to the fit of the Schade et al. (2004) microarray data.

**Fig. 11:**
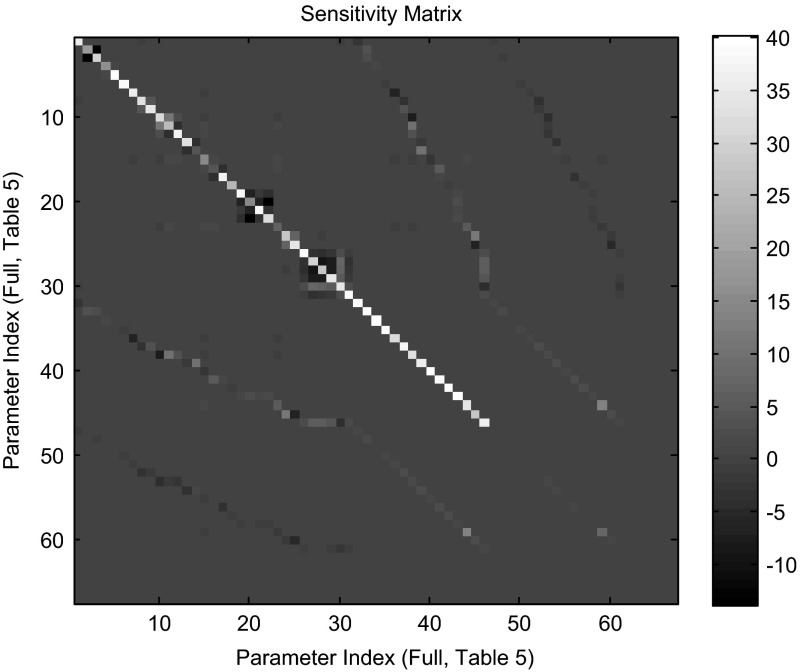
Heat map of parameter estimator vector’s covariance matrix

A final topic of interest along these lines is that of the sensitivity matrix. As discussed in Sect. 3 above, the matrix

$$\begin{aligned} V=\sum _i { \int \limits _{t_0 }^{t_f } {\frac{\partial x_i }{\partial \theta }(t,\theta ^{{*}})\frac{\partial x_i }{\partial \theta }^{T}(t,\theta ^{{*}})\mathrm{d}t} } \end{aligned}$$

measures the sensitivity of the least squares minimization to the parameters. With the parameterization under study, this matrix is of dimension $67 \times 67$. The large sample asymptotic parameter covariance matrix $\Sigma =\frac{\sigma ^{2}V^{-1}}{\sqrt{n}}$ resulting from the parameter estimation is illustrated in the heat map image of Fig. 11, which shows significant uncertainty in the weight and net threshold parameter estimates. In Fig. 11, the parameters are indexed according to the “Full Index” given in Table 5. Thus, the indices 1–31 count the weight parameters, the indices 32–46 count the net thresholds, and the indices 47–67 count the production rates (which clearly have the smallest uncertainty levels.

**Fig. 12:**
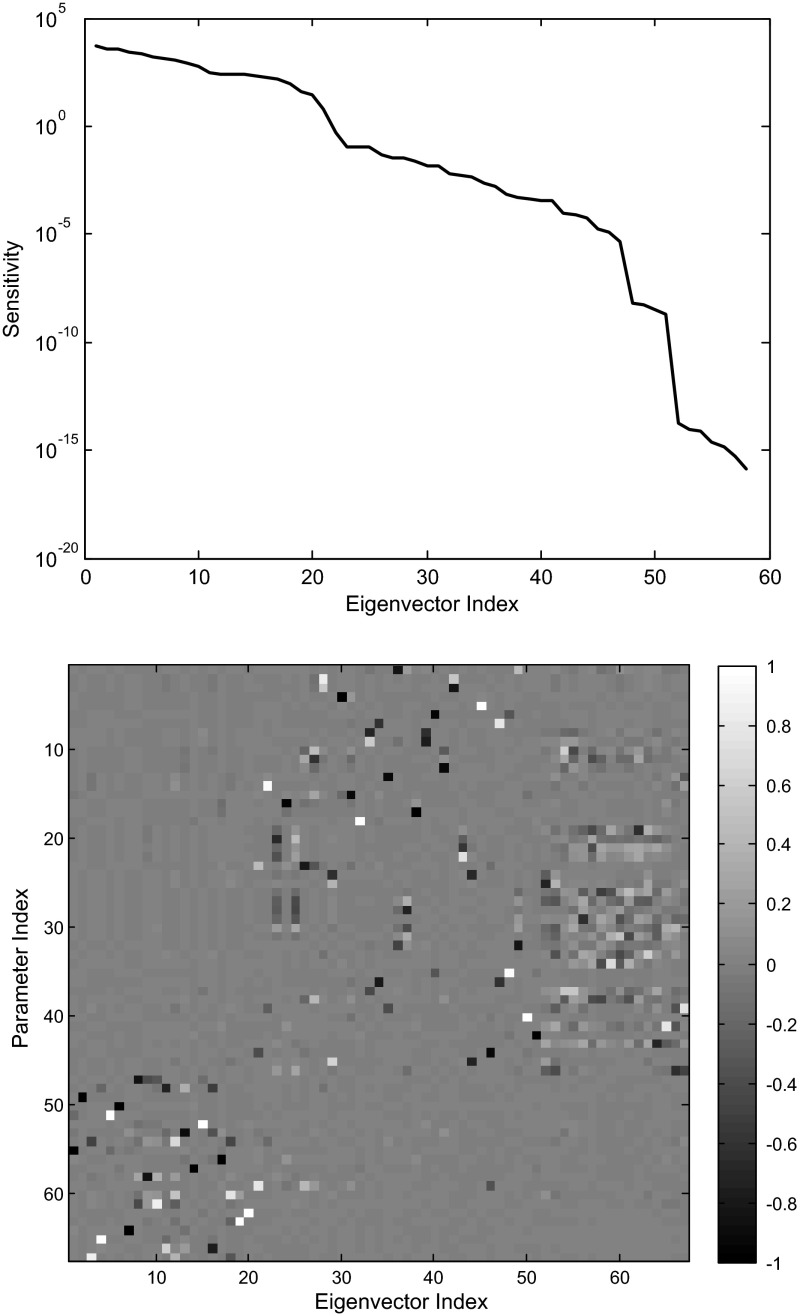
Eigenvalues (*top*) and eigenvectors (*bottom*) of the sensitivity matrix. The heat map is a three-dimensional view of the eigenvectors with intensity of the *shading* indicating the magnitude of the eigenvectors

A heat map image of the sensitivity matrix is dominated by the production rates, and the image itself is not very illuminating. In Fig. 12, we show the eigenvalues and the eigenvectors of the sensitivity matrix *V*. Some interesting patterns can be detected.

The eigenvectors in the image are ordered in terms of largest to smallest eigenvalues (that is, from highest to lowest sensitivity). The eigenvectors $$V_i $$ are ordered according to decreasing eigenvalues $$\left( {\lambda _i \ge \lambda _{i+1} } \right) $$. Note that the first 21 eigenvectors have support concentrated primarily in the production rate parameters (parameter indices 46–67, of the Full Index of Table 5), indicating that the model is most sensitive to changes in those parameters. The magnitude of the eigenvalues decreases dramatically as we move from the first 21 eigenvectors to the next 25. In this group, some interesting relationships can be observed.

**Table 4:** Network weights, net thresholds, and production rates

Edge	Weight	Standard name	*b*	*P*
ABF1 $\rightarrow$ FHL1	0.1562	ABF1	No inputs	0.4429
ABF1 $\rightarrow$ MSN1	$-$2.9707	ACE2	No inputs	0.3798
ACE2 $\rightarrow$ YAP1	$-$1.3615	AFT1	$-$0.1844	0.1712
AFT1 $\rightarrow$ AFT1	$-$0.8966	CIN5	0.8638	0.0624
CIN5 $\rightarrow$ MSN1	0.9393	CUP9	$-$0.0845	0.1052
CIN5 $\rightarrow$ ROX1	$-$0.9278	FHL1	$-$0.0270	0.0209
CIN5 $\rightarrow$ YAP6	$-$0.5312	GTS1	0.3180	0.0335
CUP9 $\rightarrow$ YAP6	$-$0.1293	HAL9	No inputs	0.0446
HAL9 $\rightarrow$ MSN4	1.4283	HSF1	2.0785	0.0396
HSF1 $\rightarrow$ REB1	$-$0.0102	MAC1	No inputs	0.0257
MAC1 $\rightarrow$ CUP9	$-$0.1882	MSN1	0.3085	0.1860
MSN4 $\rightarrow$ FHL1	0.6121	MSN4	0.5977	0.1312
NRG1 $\rightarrow$ NRG1	1.2341	NRG1	0.9144	0.2078
NRG1 $\rightarrow$ YAP6	0.6215	PHD1	No inputs	0.1302
PHD1 $\rightarrow$ CUP9	$-$0.6510	RAP1	$-$0.0836	0.0548
PHD1 $\rightarrow$ MSN4	0.5447	REB1	$-$0.1967	0.1338
RAP1 $\rightarrow$ AFT1	$-$0.4030	ROX1	$-$0.0185	0.0461
RAP1 $\rightarrow$ HSF1	$-$1.2321	RPH1	$-$1.0935	0.6910
RAP1 $\rightarrow$ MSN4	1.0131	SKN7	No inputs	0.0999
RAP1 $\rightarrow$ RAP1	$-$0.8890	YAP1	1.5146	0.1742
RAP1 $\rightarrow$ RPH1	1.4999	YAP6	0.3528	0.0790
REB1 $\rightarrow$ GTS1	0.0778			
ROX1 $\rightarrow$ YAP6	$-$0.7503			
SKN7 $\rightarrow$ NRG1	$-$0.1852			
SKN7 $\rightarrow$ ROX1	0.5744			
SKN7 $\rightarrow$ YAP1	$-$0.4082			
YAP1 $\rightarrow$ ROX1	$-$0.4315			
YAP1 $\rightarrow$ YAP6	0.0146			
YAP6 $\rightarrow$ CIN5	$-$0.0450			
YAP6 $\rightarrow$ ROX1	$-$0.5071			
YAP6 $\rightarrow$ YAP6	$-$0.3027			

The “no inputs” designation indicates that there is no regulatory influence on these genes and therefore no input value for the corresponding net threshold parameter (see Fig. 1; “Appendix”)

**Fig. 13:**
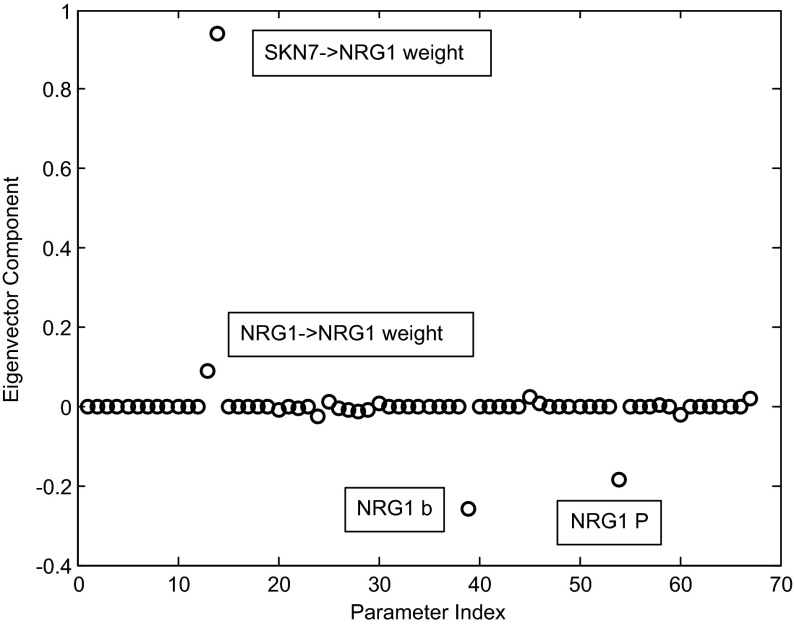
Eigenvector 22 shows the relative sensitivities in the NRG1 dynamics. The *x* axis refers to the parameter Full Index from Table 5

First, we note that Eigenvector 22 involves the state equation of NRG1. In Fig. 13, we graph Eigenvector 22, labeling the four significant parametric directions it contains.

**Fig. 14:**
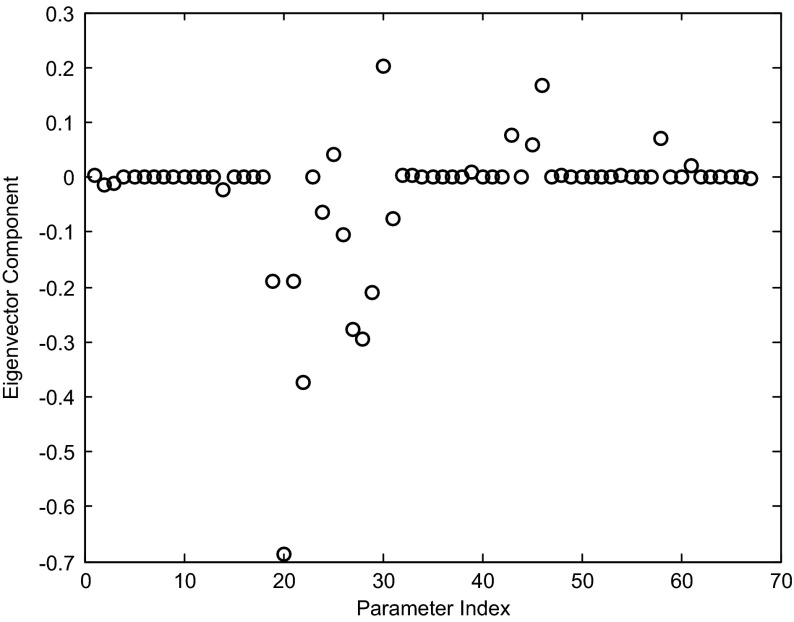
Eigenvector 23 shows the combined sensitivity to Parameters 19–22, 24–31, 43, 45, and 46. The *x* axis refers to the parameter Full Index from Table 5

The sensitivity is strongest with respect to the weight of SKN7 controlling NRG1, slightly dependent on the self-control of NRG1, with opposite sign sensitivity for the net threshold and the production rate. Eigenvector 23 shows a complex connection of sensitivities in the ROX1, YAP1, and YAP6 dynamics (Fig. 14).

The weights corresponding to the indices 19–22, 24–31 are the controlling weights for the dynamics of ROX1, YAP1, and YAP6, while indices 43, 45, and 46 correspond to the net thresholds in those three genes.

To interpret these sensitivities, we note that YAP1, ROX1, and YAP6 form a densely connected core in one sub-network (Fig. 1, upper left). Second, we observe that NRG1 is a controller of YAP6 with a fairly large positive weight (indicating activation), while four other genes controlling YAP6 are repressing, and the final sixth gene (YAP1) slightly activates YAP6 at a near-zero level almost two orders of magnitude below the activation of NRG1. Of these four genes, only ROX1 shows a significant change in gene expression ($p<0.05$ at $t_{0}$; see Table 2 and Fig. 8). The others do show fluctuations in expression (Table 2; Figs. 6, 7), but none of the $\hbox {log}_{2}$ ratios are significantly different than zero. Thus, the regulatory weights must balance each other out to conform to the observed levels of expression.

**Table 5:** Parameter indexing for Figs. 9, 10, 11, 12, 13 and 14

Weight Index	Full Index	Gene connection	b Index	Full Index	Gene	P Index	Full Index	Gene
1	1	YAP6 $\rightarrow$ CIN5	1	32	CIN5	1	47	CIN5
2	2	MAC1 $\rightarrow$ CUP9	2	33	CUP9	2	48	CUP9
3	3	PHD1 $\rightarrow$ CUP9	3	34	FHL1	3	49	FHL1
4	4	MSN4 $\rightarrow$ FHL1	4	35	GTS1	4	50	GTS1
5	5	ABF1 $\rightarrow$ FHL1	5	36	HSF1	5	51	HSF1
6	6	REB1 $\rightarrow$ GTS1	6	37	MSN1	6	52	MSN1
7	7	RAP1 $\rightarrow$ HSF1	7	38	MSN4	7	53	MSN4
8	8	CIN5 $\rightarrow$ MSN1	8	39	NRG1	8	54	NRG1
9	9	ABF1 $\rightarrow$ MSN1	9	40	RAP1	9	55	RAP1
10	10	RAP1 $\rightarrow$ MSN4	10	41	AFT1	10	56	AFT1
11	11	HAL9 $\rightarrow$ MSN4	11	42	REB1	11	57	REB1
12	12	PHD1 $\rightarrow$ MSN4	12	43	ROX1	12	58	ROX1
13	13	NRG1 $\rightarrow$ NRG1	13	44	RPH1	13	59	RPH1
14	14	SKN7 $\rightarrow$ NRG1	14	45	YAP1	14	60	YAP1
15	15	RAP1 $\rightarrow$ RAP1	15	46	YAP6	15	61	YAP6
16	16	RAP1 $\rightarrow$ AFT1				16	62	ABF1
17	17	AFT1 $\rightarrow$ AFT1				17	63	ACE2
18	18	HSF1 $\rightarrow$ REB1				18	64	HAL9
19	19	CIN5 $\rightarrow$ ROX1				19	65	MAC1
20	20	YAP1 $\rightarrow$ ROX1				20	66	PHD1
21	21	YAP6 $\rightarrow$ ROX1				21	67	SKN7
22	22	SKN7 $\rightarrow$ ROX1						
23	23	RAP1 $\rightarrow$ RPH1						
24	24	ACE2 $\rightarrow$ YAP1						
25	25	SKN7 $\rightarrow$ YAP1						
26	26	CIN5 $\rightarrow$ YAP6						
27	27	CUP9 $\rightarrow$ YAP6						
28	28	NRG1 $\rightarrow$ YAP6						
29	29	ROX1 $\rightarrow$ YAP6						
30	30	YAP1 $\rightarrow$ YAP6						
31	31	YAP6 $\rightarrow$ YAP6						

The weight indices are annotated on the edges of the network diagram in Fig. 1. The individual indices corresponding to the weights (*w*), thresholds (*b*), and production rates (*P*) are used in Figs. 9 and 10. The Full Index corresponding to the index of $\theta =\left( {w,b,P} \right)$ will be used in Figs. 11, 12, 13, and 14

**Table 6:** Standard deviations of initial guess and resulting estimates of network weights, $$w_{ij} $$, for 10 penalized least squares computations

Edge	$\sigma$ (initial guesses)	$\sigma$ (estimates)
ABF1 $\rightarrow$ FHL1	1.0763	0.000042
ABF1 $\rightarrow$ MSN1	1.0452	0.000052
ACE2 $\rightarrow$ YAP1	0.9139	0.000026
AFT1 $\rightarrow$ AFT1	1.1592	0.000016
CIN5 $\rightarrow$ MSN1	1.2506	0.000036
CIN5 $\rightarrow$ ROX1	0.7353	0.000017
CIN5 $\rightarrow$ YAP6	1.1986	0.000016
CUP9 $\rightarrow$ YAP6	0.7908	0.000022
HAL9 $\rightarrow$ MSN4	1.0100	0.000017
HSF1 $\rightarrow$ REB1	0.8139	0.000010
MAC1 $\rightarrow$ CUP9	1.0182	0.000023
MSN4 $\rightarrow$ FHL1	0.6676	0.000023
NRG1 $\rightarrow$ NRG1	1.0921	0.000033
NRG1 $\rightarrow$ YAP6	1.0962	0.000021
PHD1 $\rightarrow$ CUP9	1.3703	0.000013
PHD1 $\rightarrow$ MSN4	1.1003	0.000033
RAP1 $\rightarrow$ AFT1	0.9236	0.000003
RAP1 $\rightarrow$ HSF1	1.1732	0.000003
RAP1 $\rightarrow$ MSN4	0.6783	0.000014
RAP1 $\rightarrow$ RAP1	0.8165	0.000013
RAP1 $\rightarrow$ RPH1	0.4716	0.000007
REB1 $\rightarrow$ GTS1	0.9366	0.000006
ROX1 $\rightarrow$ YAP6	0.7266	0.000034
SKN7 $\rightarrow$ NRG1	1.1707	0.000021
SKN7 $\rightarrow$ ROX1	1.1959	0.000014
SKN7 $\rightarrow$ YAP1	0.7284	0.000006
YAP1 $\rightarrow$ ROX1	1.0836	0.000011
YAP1 $\rightarrow$ YAP6	0.7664	0.000016
YAP6 $\rightarrow$ CIN5	0.9739	0.000010
YAP6 $\rightarrow$ ROX1	0.8421	0.000033
YAP6 $\rightarrow$ YAP6	0.7260	0.000020

**Table 7:** Standard deviations of initial guess and resulting estimates of network net threshold parameters, *b* $_{i}$, for 10 penalized least squares computations

Standard name	$\sigma$ (initial guesses)	$\sigma$ (estimates)
AFT1	0.6738	0.000018
CIN5	0.9264	0.000051
CUP9	0.8543	0.000040
FHL1	1.1391	0.000026
GTS1	0.7422	0.000022
HSF1	0.8225	0.000013
MSN1	0.7975	0.000028
MSN4	0.6201	0.000013
NRG1	0.6809	0.000087
RAP1	1.2942	0.000032
REB1	1.3605	0.000028
ROX1	0.8758	0.000013
RPH1	1.2564	0.000040
YAP1	1.0017	0.000022
YAP6	0.7664	0.000012

**Table 8:** Standard deviations of initial guess and resulting estimates of production, $P_i$, rates for 10 penalized least squares computations

Standard name	$\sigma$ (initial guesses)	$\sigma$ (estimates)
ABF1	0.0182	0.000000
ACE2	0.0117	0.000000
AFT1	0.0021	0.000001
CIN5	0.0011	0.000002
CUP9	0.0014	0.000005
FHL1	0.0012	0.000001
GTS1	0.0005	0.000000
HAL9	0.0015	0.000000
HSF1	0.0019	0.000000
MAC1	0.0005	0.000000
MSN1	0.0038	0.000011
MSN4	0.0016	0.000002
NRG1	0.0033	0.000012
PHD1	0.0028	0.000000
RAP1	0.0016	0.000001
REB1	0.0030	0.000002
ROX1	0.0008	0.000001
RPH1	0.0012	0.000032
SKN7	0.0007	0.000000
YAP1	0.0014	0.000002
YAP6	0.0025	0.000002

## Concluding Remarks

We have presented a general approach to modeling medium-scale gene regulatory networks, with an emphasis on the ability to extract parameters from data obtained from microarray experiments. Our findings are that a high-dimensional parameter vector in a complex high-dimensional dynamic network model can be reliably inferred from temporally sparse microarray data using a penalized least squares approach. The resulting dynamics are not, however, calibrated to units of concentration in mass balance, due to the relative nature of two-color microarray measurement technology. Furthermore, our model does not separate rates of mRNA and protein production or degradation. The model starts with a network topology and extracts relative strength of relationships, direction (activation/repression) of relationships, and rate of expression. The magnitude of the parametric uncertainties, as measured through the covariance, are large enough to preclude the use of this approach in extracting the network topology from data at this coarse level of time resolution, so the techniques described herein must be used in conjunction with other methods, either statistical clustering approaches or additional experiments, to identify the network connections. We are confident, however, in the utility of this approach to refine the dynamics and directionality of a candidate regulatory graph, which should have general applicability to other biological problems where time course gene expression data are available.

Biologically, the estimated model parameters have shed light on the regulation of the early transcriptional response to cold shock in *S. cerevisiae* for which we had three questions: (1) which transcription factors control the early response to cold shock in *S. cerevisiae*? (2) what is the extent of ESR pathway overlap? (3) which part of the transcriptional response to cold shock is due to indirect effects of other transcription factors? First, the Schade et al. (2004) expression data and inferred network weights (Tables 2, 4) suggest that the subnetwork of transcription factors centered around RAP1 and including FHL1, MSN4, RPH1, and HSF1 plays a prominent role in the regulation of the cold shock response (Fig. 8, lower right). This makes sense biologically because RAP1 and FHL1 are responsible for activating genes encoding ribosomal proteins, and ribosome biogenesis is a biological process known to be induced by cold shock (Aguilera et al. 2007; Xiao and Grove 2009). RAP1 acts as both an activator and repressor in the model and is known to have both transcriptional activator and repressor activity in the cell (Shore and Nasmyth 1987). RAP1 strongly activates MSN4 and RPH1 in our model, both of which have significant changes in gene expression in the Schade et al. (2004) data. Indeed, all three inputs to MSN4 activate it. Both MSN4 and RPH1 bind to stress response elements (STRE) in approximately 200 genes, the activation of which constitutes the general ESR (Gasch et al. 2000; Causton et al. 2001; Orzechowski et al. 2012). FHL1 is weakly activated by both MSN4 and ABF1. Because ABF1 itself is down-regulated, the main activating influence comes from MSN4. However, FHL1 itself is down-regulated, so there must be another transcription factor outside this network that influences its expression. RAP1 also strongly represses HSF1, which is significantly downregulated in expression. HSF1 is responsible for inducing genes required for the heat shock response (Morano et al. 2012). There is some evidence to suggest that the cold shock response has some “opposite” effects than the heat shock response, so the down-regulation of HSF1 makes sense (Gasch et al. 2000; Schade et al. 2004). Thus, our model indicates that further examination of the roles of RAP1, FHL1, MSN4, RPH1, and HSF1 in regulating the early response to cold shock is warranted.

In contrast, the other subnetwork, (upper left of Fig. 8, including ACE2, CIN5, MSN1, NRG1, ROX1, SKN7, YAP1, and YAP6) appears to play less of a role in controlling the early cold shock response as there are few significant changes in gene expression in that part of the network. If the weights of the incoming edges are summed for each gene, they are all negative except for the weights controlling NRG1. Even though the weights of CIN5 and ABF1 controlling MSN1 are among the largest in magnitude in the entire network, they have opposite effects. CIN5 strongly activates MSN1, while ABF1 strongly represses it with the sum of the weights being negative; however, from the data, we see that the expression of MSN1 is unchanged.

Second, in terms of ESR pathway overlap, RAP1, FHL1, MSN4, RPH1, and HSF1 have all been implicated in controlling the response to other environmental stresses (Gasch et al. 2000; Causton et al. 2001; Morano et al. 2012; Orzechowski et al. 2012; Xiao and Grove 2009). Our model suggests that there is overlap between the general ESR and the early response to cold, not just the late cold response as noted in Schade et al. (2004) and Kandror et al. (2004).

Third, as for the indirect effects of transcription factors, as noted in Sect. 2, our network has regulatory chains that are 4 or 5 nodes deep and two complex feedforward motifs. However, it appears that the influence of transcription factors in a regulatory chain peters out after just one or two nodes. For example, RAP1 strongly influences HSF1 and MSN4, but the influence of HSF1 upon REB1 and MSN4 upon FHL1 are much weaker. Furthermore, as has already been noted, there is evidence to suggest that additional transcription factors not included in our network are necessary to explain the expression of the genes in our network. For example, RAP1 is found to repress itself in the model, even though it shows a significant increase in expression after 120 min of cold exposure, so there must be another transcription factor activating it that was not included in this network. FHL1 is significantly downregulated in expression, but its regulators ABF1 and MSN4 only weakly activate it, suggesting that FHL1, too, is repressed by an additional factor outside the current model. The significant down-regulation in the expression of ABF1 in the data, together with the fact that there are no predicted gene regulators for ABF1 in the current network, suggests that this must be due to some other transcription factors outside this network. Finally, MAC1 also shows a significant increase in gene expression at the $t_{120}$ timepoint, but is also not regulated by any transcription factors in the current network, necessitating the invocation of other regulators.

The results of this model suggest several lines of future investigation, both experimentally and computationally. The model highlights the role of RAP1, FHL1, MSN4, RPH1, and HSF1 in regulating the early response to cold shock. A natural next experiment would be to investigate how the early response to cold shock is affected by the deletion of those genes. Unfortunately, RAP1, HSF1, and FHL1 are all essential genes in yeast, making the simple knockout experiment impossible (Winzeler et al. 1999). However, MSN4 and RPH1 are not essential and could be investigated in such a way. Although Schade et al. (2004) did perform microarray experiments on a strain deleted for both the MSN2 and MSN4 transcription factors, they only performed two replicates with the double deletion strain, precluding statistical analysis of the data that would indicate its reliability for use in estimating model parameters, and leaving additional experiments to be performed. Such biological knockout experiments could then be complemented by *in silico* knockouts where parameter estimation and forward simulations are performed using networks with the appropriate transcription factors removed. A comparison of the experimental and computational results could lead to refinements of the model and further biological insights. However, given that it appears that ABF1, FHL1, MAC1, and RAP1 are regulated by transcription factors not included in our network, a new network would need to be defined that includes those potential regulating factors. To our knowledge, genome-wide location analysis has not been performed under cold shock conditions, so important network connections could be missing from the currently available experimental data, necessitating other approaches for defining the regulatory network.

In conclusion, we have successfully estimated model parameters from microarray data for a medium-scale gene regulatory network using a penalized least squares approach. The results accurately model the expression dynamics, have revealed activation and repression relationships between the transcription factors in our network, and suggest which factors are most important to the regulation of the early response to cold shock in *S. cerevisiae*. Our work provides a firm mathematical foundation and specific biological suggestions with testable hypotheses for future systems biology iterations of modeling and experiment regarding the cold shock response in yeast. Finally, our work has general applicability to other biological systems.

### Electronic supplementary material

Supplementary material 1 (zip 65 KB)

## Appendix

See Table 9.

**Table 9:** List of transcription factors included in the gene regulatory network model

Standard name	Systematic name	Alias	Input TFs	Output TFs
ABF1	YKL112W	BAF1, OBF1, REB2, SBF1		FHL1, MSN1
ACE2	YLR131C			YAP1
AFT1	YGL071W	RCS1	AFT1, RAP1	AFT1
CIN5	YOR028C	HAL6, YAP4	YAP6	MSN1, ROX1, YAP6
CUP9	YPL177C		MAC1, PHD1	YAP6
FHL1	YPR104C	SPP42	ABF1, MSN4	
GTS1	YGL181W	FHT1, LSR1	REB1	
HAL9	YOL089C			MSN4
HSF1	YGL073W	EXA3, MAS3	RAP1	REB1
MAC1	YMR021C	CUA1		CUP9
MSN1	YOL116W	FUP1, HRB382, MSS10, PHD2	ABF1, CIN5	
MSN4	YKL062W		HAL9, PHD1, RAP1	
NRG1	YDR043C		NRG1, SKN7	NRG1, YAP6
PHD1	YKL043W			CUP9, MSN4
RAP1	YNL216W	GRF1, TBA1, TUF1	RAP1	AFT1, HSF1, MSN4, RAP1, RPH1
REB1	YBR049C	GRF2	HSF1	GTS1
ROX1	YPR065W	REO1	CIN5, SKN7, YAP1, YAP6	YAP6
RPH1	YER169W		RAP1	
SKN7	YHR206W	BRY1, POS9		NRG1, ROX1, YAP1
YAP1	YML007W	PAR1, SNQ3	ACE2, SKN7	ROX1, YAP6
YAP6	YDR259C	HAL7	CIN5, CUP9, NRG1, ROX1, YAP1, YAP6	CIN5, ROX1, YAP6

## References

[CR1] Ackleh AS, Fitzpatrick BG, Scribner R, Simonsen N, Thibodeaux JJ (2009). Ecosystem modeling of college drinking: parameter estimation and comparing models to data. Math Comput Model.

[CR2] Aguilera J, Randez-Gil F, Prieto JA (2007). Cold response in *Saccharomyces cerevisiae*: new functions for old mechanisms. FEMS Microbiol Rev.

[CR3] Al-Fageeh MB, Smales CM (2006). Control and regulation of the cellular responses to cold shock: the responses in yeast and mammalian systems. Biochem J.

[CR4] Alon U (2007). An introduction to systems biology: design principles of biological circuits.

[CR5] Angeli D, Hirsch MW, Sontag ED (2009). Attractors in coherent systems of differential equations. J Differ Equ.

[CR6] Bailey KR, Fitzpatrick BG (1997). Estimation of groundwater flow parameters using least squares. Math Comput Model.

[CR7] Banks HT, Fitzpatrick BG (1990). Statistical methods for model comparison in parameter estimation problems for distributed systems. J Math Biol.

[CR8] Belle A, Tanay A, Bitincka L, Shamir R, O’Shea EK (2006). Quantification of protein half-lives in the budding yeast proteome. Proc Natl Acad Sci USA.

[CR9] Berger JO (1993). Statistical decision theory and Bayesian analysis.

[CR10] Blossey R, Cardelli L, Phillips A (2008). Compositionality, stochasticity, and cooperativity in dynamic models of gene regulation. HFSP J.

[CR11] Cao J, Zhao H (2008). Estimating dynamic models for gene regulation networks. Bioinformatics.

[CR12] Causton HC, Ren B, Koh SS, Harbison CT, Kanin E, Jennings EG, Lee TI, True HL, Lander ES, Young RA (2001). Remodeling of yeast genome expression in response to environmental changes. Mol Biol Cell.

[CR13] Chen K-C, Wang T-Y, Tseng H-H, Huang C-YF, Kao C-Y (2005). A stochastic differential equation model for quantifying transcriptional regulatory network in *Saccharomyces cerevisiae*. Bioinformatics.

[CR14] Chen T, He HL, Church GM (1999). Modeling gene expression with differential equations. Pac Symp Biocomput.

[CR15] Climescu-Haulica A, Quirk MD (2007). A stochastic differential equation model for transcriptional regulatory networks. BMC Bioinf.

[CR16] Dawes IW, Dickinson JR, Schweizer M (2004). Stress responses. The metabolism and molecular physiology of *Saccharomyces cerevisiae*.

[CR17] Fan M, Kuwahara H, Wang X, Wang S, Gao X (2015) Parameter estimation methods for gene circuit modeling from time-series mRNA data: a comparative study. Brief Bioinf bbv015. doi:10.1093/bib/bbv015 10.1093/bib/bbv015 25818863

[CR18] Fitzpatrick BG (1993). Parameter estimation in conservation laws. J Math Syst Est Control.

[CR19] Fitzpatrick BG (2008). Statistical considerations and techniques for understanding physiological data, modeling, and treatments. Cardiovasc Eng.

[CR20] Fitzpatrick BG, Keeling SL (1997). On approximation in total variation penalization for image reconstruction and inverse problems. Numer Func Anal Opt.

[CR21] Gallant AR (1987). Nonlinear statistical models.

[CR22] Gasch AP, Spellman PT, Kao CM, Carmel-Harel O, Eisen MB, Storz G, Botstein D, Brown PO (2000). Genomic expression programs in the response of yeast cells to environmental changes. Mol Biol Cell.

[CR23] Golub GH, Heath M, Wahba G (1979). Generalized cross-validation as a method for choosing a good ridge parameter. Technometrics.

[CR24] Hansen PC, O’Leary DP (1993). The use of the L-curve in the regularization of discrete ill-posed problems. SIAM J Sci Comput.

[CR25] Harbison CT, Gordon DB, Lee TI, Rinaldi NJ, Macisaac KD, Danford TW, Hannett NM, Tagne JB, Reynolds DB, Yoo J, Jennings EG, Zeitlinger J, Pokholok DK, Kellis M, Rolfe PA, Takusagawa KT, Lander ES, Gifford DK, Fraenkel E, Young RA (2004). Transcriptional regulatory code of a eukaryotic genome. Nature.

[CR26] Hecker M, Lambeck S, Toepfer S, Van Someren E, Guthke R (2009). Gene regulatory network inference: data integration in dynamic models—a review. Biosystems.

[CR27] Huet S, Bouvier A, Poursat M-A, Jolivet E (2004). Statistical tools for nonlinear regression: a practical guide with S-PLUS and R examples.

[CR28] Kandror O, Bretschneider N, Kreydin E, Cavalieri D, Goldberg AL (2004). Yeast adapt to near-freezing temperatures by STRE/Msn2,4-dependent induction of trehalose synthesis and certain molecular chaperones. Mol Cell.

[CR29] Karlebach G, Shamir R (2008). Modelling and analysis of gene regulatory networks. Nat Rev Mol Cell Biol.

[CR30] Kauffman KJ, Prakash P, Edwards JS (2003). Advances in flux balance analysis. Curr Opin Biotechnol.

[CR31] Kuwahara H, Fan M, Wang S, Gao X (2013). A framework for scalable parameter estimation of gene circuit models using structural information. Bioinformatics.

[CR32] Lee TI, Rinaldi NJ, Robert F, Odom DT, Bar-Joseph Z, Gerber GK, Hannett NM, Harbison CT, Thompson CM, Simon I, Zeitlinger J, Jennings EG, Murray HL, Gordon DB, Ren B, Wyrick JJ, Tagne JB, Volkert TL, Fraenkel E, Gifford DK, Young RA (2002). Transcriptional regulatory networks in *Saccharomyces cerevisiae*. Science.

[CR33] Lillacci G, Khammash M (2010). Parameter estimation and model selection in computational biology. PLoS Comput Biol.

[CR34] Mendoza L, Xenarios I (2006). A method for the generation of standardized qualitative dynamical systems of regulatory networks. Theor Biol Med Model.

[CR35] Morano KA, Grant CM, Moye-Rowley WS (2012). The response to heat shock and oxidative stress in *Saccharomyces cerevisiae*. Genetics.

[CR36] Murata Y, Homma T, Kitagawa E, Momose Y, Sato MS, Odani M, Shimizu H, Hasegawa-Mizusawa M, Matsumoto R, Mizukami S, Fujita K, Parveen M, Komatsu Y, Iwahashi H (2006). Genome-wide expression analysis of yeast response during exposure to 4 degrees C. Extremophiles.

[CR37] Orzechowski Westholm J, Tronnersjö S, Nordberg N, Olsson I, Komorowski J, Ronne H (2012). Gis1 and Rph1 regulate glycerol and acetate metabolism in glucose depleted yeast cells. PLoS ONE.

[CR38] Sahara T, Goda T, Ohgiya S (2002). Comprehensive expression analysis of time-dependent genetic responses in yeast cells to low temperature. J Biol Chem.

[CR39] Schade B, Jansen G, Whiteway M, Entian KD, Thomas DY (2004). Cold adaptation in budding yeast. Mol Biol Cell.

[CR40] Schlitt T, Brazma A (2007). Current approaches to gene regulatory network modelling. BMC Bioinf.

[CR41] Shea MA, Ackers GK (1985). The OR control system of bacteriophage lambda. A physical–chemical model for gene regulation. J Mol Biol.

[CR42] Shore D, Nasmyth K (1987). Purification and cloning of a DNA binding protein from yeast that binds to both silencer and activator elements. Cell.

[CR43] Smolen P, Baxter DA, Byrne JH (2000). Modeling transcriptional control in gene networks-methods, recent results, and future directions. Bull Math Biol.

[CR44] Sontag ED (2007). Monotone and near-monotone biochemical networks. Syst Synth Biol.

[CR45] Stekel D (2003). Microarray bioinformatics.

[CR46] Tai SL, Daran-Lapujade P, Walsh MC, Pronk JT, Daran J-M (2007). Acclimation of *Saccharomyces cerevisiae* to low temperature: a chemostat-based transcriptome analysis. Mol Biol Cell.

[CR47] Tang L, Liu X, Clarke ND (2006). Inferring direct regulatory targets from expression and genome location analyses: a comparison of transcription factor deletion and overexpression. BMC Genom.

[CR48] Thieringer HA, Jones PG, Inouye M (1998) Cold shock and adaptation. Bioessays 20:49–57. doi: 10.1002/(SICI)1521-1878(199801)20:1$$<$$3.0.CO;2-N 10.1002/(SICI)1521-1878(199801)20:1<49::AID-BIES8>3.0.CO;2-N 9504047

[CR49] Vohradský J (2001). Neural network model of gene expression. FASEB J.

[CR50] Vu TT, Vohradsky J (2007). Nonlinear differential equation model for quantification of transcriptional regulation applied to microarray data of Saccharomyces cerevisiae. Nucleic Acids Res.

[CR51] Wilkinson DJ (2006). Stochastic modelling for systems biology.

[CR52] Winzeler EA, Shoemaker DD, Astromoff A, Liang H, Anderson K, Andre B, Bangham R, Benito R, Boeke JD, Bussey H, Chu AM, Connelly C, Davis K, Dietrich F, Dow SW, El Bakkoury M, Foury F, Friend SH, Gentalen E, Giaever G, Hegemann JH, Jones T, Laub M, Liao H, Liebundguth N, Lockhart DJ, Lucau-Danila A, Lussier M, M’Rabet N, Menard P, Mittmann M, Pai C, Rebischung C, Revuelta JL, Riles L, Roberts CJ, Ross-MacDonald P, Scherens B, Snyder M, Sookhai-Mahadeo S, Storms RK, Véronneau S, Voet M, Volckaert G, Ward TR, Wysocki R, Yen GS, Yu K, Zimmermann K, Philippsen P, Johnston M, Davis RW (1999). Functional characterization of the *S. cerevisiae* genome by gene deletion and parallel analysis. Science.

[CR53] Xiao L, Grove A (2009). Coordination of ribosomal protein and ribosomal RNA gene expression in response to TOR signaling. Curr Genomics.

