# Supplementary material for: Parameter Estimation for Gene Regulatory Networks from Microarray Data: Cold Shock Response in Saccharomyces cerevisiae
Source: Bull Math Biol. 2015 Sep 29;77(8):1457–92. doi: 10.1007/s11538-015-0092-6 (PMC4636536; doi:10.1007/s11538-015-0092-6)
Supplement: Supplementary file 1 — Supplementary material 1 (zip 65 KB) [file 11538_2015_92_MOESM1_ESM.zip › Dahlquist_et_al_2015_supplementary_material_readme.pdf]

ReadMe for Supplementary Material

for Dahlquist KD, Fitzpatrick BG, Camacho ET, Entzminger SD, Wanner NC (2015) Parameter Estimation for Gene Regulatory Networks from Microarray Data: Cold Shock Response in *Saccharomyces cerevisiae.* Bull Math Biol. doi: 10.1007/s11538-015-0092-6

The file `21-genes_31-edges_Schade-data_input.xlsx` is the input Excel Workbook for the MATLAB model and the file `21-genes_31-edges_Schade-data_estimation_output.xlsx` is the resulting output file that corresponds to the data and figures in the paper. The contents of each worksheet for each file are detailed below:

21-genes_31-edges_Schade-data_input.xlsx

production_rates: Initial guesses for the production rate parameters, *P*. Assuming that the system is in steady state with the relative expression of all genes equal to 1, $\frac{P}{2} - \lambda = 0$, where λ is the degradation rate, is a reasonable initial guess.

degradation_rates: Degradation rates based on published protein half-life data from Belle et al. (2006). We converted the half-life data values to the degradation rates by taking the natural log of the half-life and dividing by 2. These values are presented in Table 3 in the manuscript.

log2_concentrations: Average $\log_2$ ratios of expression derived from Schade et al. (2004) described in Section 3 and presented in Table 2 of the manuscript. These values also correspond to the green circles in Figures 5, 6, and 7.

concentration_sigmas: Standard deviations of the $\log_2$ ratios of expression derived from Schade et al. (2004). These values correspond to the red crosses in Figures 5, 6, and 7 in the manuscript.

network: Adjacency matrix representation of the gene regulatory network. The columns correspond to the transcription factors and the rows correspond to the target genes controlled by those transcription factors. A “1” means there is an edge connecting them and a “0” means that there is no edge connecting them. This network is depicted in Figure 1 of the manuscript.

network_weights: The same format as the “network” sheet above. These are the initial guesses for the estimation of the *w* parameters.

network_thresholds: The same format as the “network” sheet above. These are the initial guesses for the estimation of the τ parameters on page 13 of the manuscript. The user chooses which threshold parameterization to use (*b* or τ) in the optimization parameters sheet below.

optimization_parameters:

- **b_or_tau:** indicator switch: 1 to estimate *b*, 0 to estimate τ
- **alpha:** Penalty term weighting (as in the L-curve analysis of Figure 4 in the manuscript)
- **kk_max:** Number of times to re-run the optimization loop: in some cases re-starting the optimization loop can improve performance of the estimation.
- **MaxIter:** Number of times MATLAB iterates through the optimization scheme
- **TolFun:** how close successive least squares cost evaluations should be before MATLAB determines that it is not making any improvement
- **MaxFunEval:** maximum number of times MATLAB will evaluate the least squares cost

TolX: how close successive parameter estimates need to be before MATLAB determines that it is not making any improvement

simulation_times: timepoints (in minutes) at which to evaluate the differential equations in the forward simulation.

network_b: The same format as the "production_rates" sheet above. These are the initial guesses for the estimation of the *b* parameters on page 16 of the manuscript.

`21-genes_31-edges_Schade-data_estimation_output.xlsx`

Sheet1: Empty sheet, created when the output sheet is created in MATLAB.

Sheet2: Empty sheet, created when the output sheet is created in MATLAB.

Sheet3: Empty sheet, created when the output sheet is created in MATLAB.

log2_concentrations: Average $\log_2$ ratios of expression derived from Schade et al. (2004) described in Section 3 and presented in Table 2 of the manuscript. These values also correspond to the green circles in Figures 5, 6, and 7. Identical to input sheet.

log2_optimized_concentrations: Model-derived $\log_2$ ratios of expression from a forward simulation using the optimized parameters and the simulation times listed in the input sheet. These values correspond to the solid blue curves in Figures 5, 6, and 7 in the manuscript.

degradation_rates: Degradation rates based on published protein half-life data from Belle et al. (2006). We converted the half-life data values to the degradation rates by taking the natural log of the half-life and dividing by 2. These values are presented in Table 3 in the manuscript. Identical to input sheet.

production_rates: Optimized production rate parameters, *P*, presented in Table 4 of the manuscript

measurement_times: Timepoints (minutes) measured in the Schade et al. (2004) microarray data.

network: Adjacency matrix representation of the gene regulatory network. The columns correspond to the transcription factors and the rows correspond to the target genes controlled by those transcription factors. A "1" means there is an edge connecting them and a "0" means that there is no edge connecting them. This network is depicted in Figure 1 of the manuscript. Identical to input sheet.

network_weights: The same format as the "network" sheet above. These are the initial guesses for the estimation of the *w* parameters. Identical to the input sheet.

network_optimized_b: Optimized *b* parameters, corresponding to Table 4 of the manuscript.

network_optimized_weights: The same format as the "network" sheet above. Optimized weight parameters, *w*, corresponding to Table 4 and depicted in Figure 8 of the manuscript.

concentration_sigmas: Standard deviations of the $\log_2$ ratios of expression derived from Schade et al. (2004). These values correspond to the red crosses in Figures 5, 6, and 7 in the manuscript. Identical to input sheet.

References

Belle A, Tanay A, Bitincka L, Shamir R, O'Shea EK (2006) Quantification of protein half-lives in the budding yeast proteome. Proc Natl Acad Sci U S A 103:13004–13009. doi:10.1073/pnas.0605420103

Schade B, Jansen G, Whiteway M, Entian KD, Thomas DY (2004) Cold adaptation in budding yeast. Mol Biol Cell 15:5492–5502. doi:10.1091/mbc.E04-03-0167
